# A glycaemic index compendium of non-western foods

**DOI:** 10.1038/s41387-020-00145-w

**Published:** 2021-01-06

**Authors:** Christiani Jeyakumar Henry, Rina Yu Chin Quek, Bhupinder Kaur, Sangeetha Shyam, Harvinder Kaur Gilcharan Singh

**Affiliations:** 1 Singapore Institute of Food and Biotechnology Innovation (SIFBI), Clinical Nutrition Research Centre (CNRC), 14 Medical Drive, #07-02, Singapore, 117599 Singapore; 2 Department of Biochemistry, National University of Singapore (NUS), 8 Medical Drive, Singapore, 117596 Singapore; 3 Division of Nutrition and Dietetics, School of Health Sciences, International Medical University (IMU), No. 126, Jln Jalil Perkasa 19, Bukit Jalil 57000 Kuala Lumpur, Malaysia; 4 Centre for Translational Research, Institute for Research, Development and Innovation (IRDI), International Medical University (IMU), No. 126, Jln Jalil Perkasa 19, Bukit Jalil 57000 Kuala Lumpur, Malaysia; 5 Centre for Environmental and Population Health, Institute for Research, Development and Innovation (IRDI), International Medical University (IMU), No. 126, Jln Jalil Perkasa 19, Bukit Jalil 57000 Kuala Lumpur, Malaysia

**Keywords:** Health care, Signs and symptoms

## Abstract

Current international tables published on the glycaemic index (GI) of foods represent valuable resources for researchers and clinicians. However, the vast majority of published GI values are of Western origin, notably European, Australian and North American. Since these tables focus on Western foods with minimal inclusion of other foods from non-Western countries, their application is of limited global use. The objective of this review is to provide the GI values for a variety of foods that are consumed in non-Western countries. Our review extends and expands on the current GI tables in an attempt to widen its application in many other regions of the world.

## Introduction

In many non-Western countries, cereal-based carbohydrates provide ~60% of total energy intake^1^ compared with 42% for Caucasians^2^. The consumption of such high-carbohydrate diets yield high glucose and insulin response, thus contributing to insulin resistance. Nonetheless, the quality of carbohydrate consumed is as important as the quantity. Poor quality carbohydrates are quickly digested and absorbed, thereby giving rise to high blood glucose and insulin ‘spikes’. Observational studies have shown that the consumption of low glycaemic index (GI) foods is associated with a lower risk of type 2 diabetes mellitus (T2DM)^3^, significantly less insulin resistance and a lower prevalence of the metabolic syndrome^4^. However, the vast majority of these published GI values are of European, Australian and North America origin.

## Glycaemic index

The GI is defined as a numerical figure used to represent the ability of a carbohydrate food to raise blood glucose levels. It is expressed as a percentage of the incremental area under the glycaemic response curve (AUC) elicited by a portion of food containing 50 g available carbohydrate in comparison with the AUC elicited by a standard reference food of 50 g glucose or white bread in the same participant^5^. The principle is that the slower the rate of carbohydrate absorption into the bloodstream, the lower the rise of blood glucose level and the lower the GI value. A GI value of ≥70 is considered high, a GI value 56–69 inclusive is medium and a GI value ≤55 is low, where glucose = 100.

Following the approach of these authors, we are for the first time providing a compendium of GI values of non-Western foods^6^. Since many of these GI values were published in uncommon journals or located in various sources, it is not surprising that many previous authors may have found it a challenge to access and retrieve such information.

With a global pandemic of T2DM escalating, especially in emerging countries^7^, it is now recognised that the GI food-based intervention is an important tool in the management and prevention of T2DM^8^. Ironically, in regions of the world where there is a pandemic of T2DM, there is a shortage of a taxonomy of GI data of non-Western foods (e.g. Middle East, South Asia, Indian sub-continent) in contrast to the current international GI tables^6,9^.

In 1997, the FAO/WHO Expert Consultation suggested that the concept of GI might provide a useful means of helping to select the most appropriate carbohydrate-containing foods for the maintenance of health and the treatment of several diseases^10^. A meta-analysis by Brand-Miller et al^11^. demonstrated that choosing low GI foods in place of conventional or high GI foods exhibited a small but clinically important effect on medium-term glycaemic control in patients with diabetes. Low GI foods have been shown to reduce insulin demand and lipid concentrations, improve blood glucose control and reduce body weight, thus preventing diabetes-related cardiovascular events^12–15^.

A typical non-Western diet, such as in South Asia, is high in carbohydrates with cereals such as polished rice, white flour, finger millet, semolina and wheat providing the bulk of the energy^16^. Furthermore, it has been shown that a unique metabolic feature of South Asians, for an identical carbohydrate load, elicits postprandial glucose peaks that are 2–3 times larger than Caucasians^17–19^. Hence, a compilation of the GI of non-Western foods is necessary for proper selection and modifications that may be of particular benefit to not only these groups of people but to a wider audience.

The objective of this review is, therefore, to consolidate the GI values for a variety of foods that are consumed in non-Western countries. This is in order to capture and encapsulate all the data available on GI that have not been reported in the general literature. Given that the largest preponderance of type 2 diabetes is in Asia, the Middle East, South America and parts of Africa, it is imperative that the database on GI is expanded in order for it to have global utility. With this in mind, papers were critically evaluated based on a strict criterion. The emphasis of this review has inevitably been to record and document the GI of various foods.

### Research design and method

We conducted a comprehensive literature search for relevant, original articles published from January 2000 through May 2020. Since 2000 marked the exponential growth in GI testing in non-Western countries, we have decided to take this as the year of data analysis. Briefly, the following string of search terms was used in PubMed and Google Scholar, with no language or other restrictions: (glycaemic index) AND (foods) AND (‘country’). The electronic search was supplemented by manual searches through the reference sections of selected publications, as well as with linked articles that were found to have cited these particular publications. Non-Western countries included in this search were as follows: Singapore, Malaysia, Indonesia, Brunei, Cambodia, Thailand, Japan, Korea, China, Taiwan, Hong Kong, Nepal, India, Myanmar, Vietnam, Sri Lanka, Philippines, United Arab Emirates, Yemen, Oman, Saudi, Qatar, Kuwait, Lebanon, Egypt, Pakistan and Bangladesh. The compiled GI studies in our article have utilised the recommended GI testing method^10,20^ and fulfilled the minimum requirements for the following inclusion criteria for GI testing studies: minimum 10 participants (healthy/T2DM), instruments such as Yellow Spring Instruments (YSIs) and the use of handheld glucometers such as HemoCue® and other similar devices widely used in clinical studies for GI testing, amount of available carbohydrate and reference food (glucose/white bread/white rice). In the table, most of the foods are based on 50 g available carbohydrate. However, for foods with low to moderate carbohydrate density, it is justified by Brouns et al.^20^ to use a lower amount of carbohydrate to prevent consumption of an excessively large amount of food. Finally, the GI of non-Western foods were grouped according to the countries. The food list was arranged according to the country of origin so as to allow individuals who are keener on knowing the GI variability of foods from their own country to retrieve this information conveniently.

## Results

**Fig. 1:**
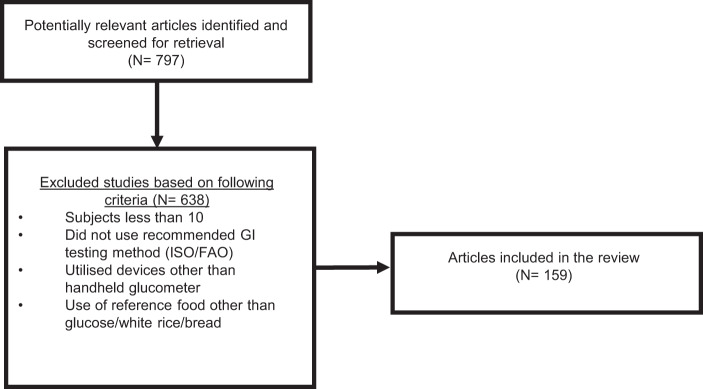
Flowchart illustrating number of studies screened, excluded and included. N - number of studies, ISO - International Organisation for Standardisation, FAO - Food and Agriculture Organisation.

**Fig. 2:**
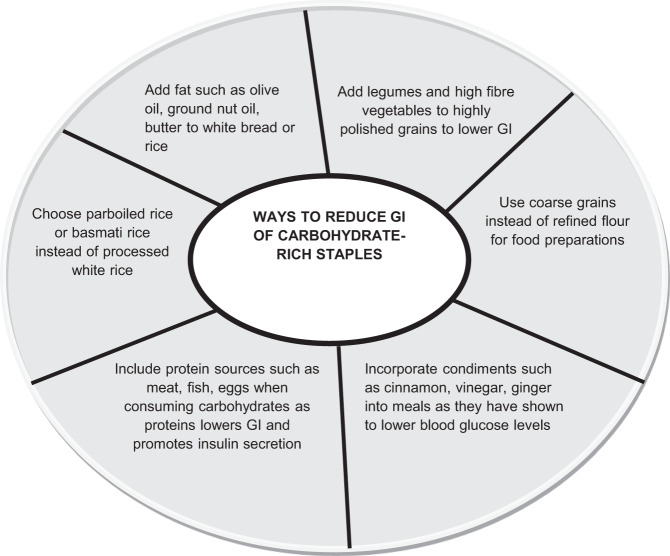
Recommendations of ways to reduce the GI of carbohydrate-rich staples. Figure shows how combination of food ingredients and foods may be used to reduce the glycaemic response of rice-based staples.

Table 1 lists 940 food items, citing 159 separate studies, representing reliable data derived from healthy subjects or individuals with type 2 diabetes. Figure 1 shows a flow diagram indicating a number of studies screened, excluded and included in this article. Non-Western countries included in this compilation were as follows: Singapore, Malaysia, Thailand, Indonesia, Philippines, Japan, Korea, China, Taiwan, Hong Kong, India, Sri Lanka, Emirates, Oman, Saudi and Lebanon. These countries were chosen based on published studies on GI from these locations, with validated methodologies used and the papers followed the inclusion criteria as described in our paper. The GI of non-Western foods was firstly arranged according to the country it was derived from. This was followed by the food item, the GI, serving size (if any), available carbohydrate portion, subject type and number, and lastly the reference food with time period of GI testing. An important feature of our paper is to encourage readers to interpret the data in a way that will enable them to select healthy foods from the GI range of foods available. Therefore, using the data generated from our GI tables, the illustrative example presented below are simple methods that may be adapted to reduce the GI values of carbohydrate-rich staples (Fig. 2).

**Table 1:** GI values of non-Western foods.

	Reference	Country	Food item	GI (glucose = 100)		Serving size (g) per experimental portion size	Available CHO (g) per experimental portion	Participants (type and number)	Reference food and time period
				Mean	SEM				
1	^21^	Singapore	Malay-style fried rice	99	7	377	50	11 Healthy participants	Glucose/2 h
2	^21^	Singapore	Nasi lemak	100	14	210	50	11 Healthy participants	Glucose/2 h
3	^21^	Singapore	Mee goreng	91	9	309	50	11 Healthy participants	Glucose/2 h
4	^21^	Singapore	Mee siam	88	11	655	50	11 Healthy participants	Glucose/2 h
5	^21^	Singapore	Loi mai kai	94	9	149	50	11 Healthy participants	Glucose/2 h
6	^21^	Singapore	Red bean pau	91	6	67	50	11 Healthy participants	Glucose/2 h
7	^21^	Singapore	Chicken Curry Puff	92	8	71	50	11 Healthy participants	Glucose/2 h
8	^21^	Singapore	Cheese bun	95	9	52	50	11 Healthy participants	Glucose/2 h
9	^22^	Singapore	White rice	96	6.6	194 g cooked rice	50	12 Healthy participants	Glucose/2 h
10	^22^	Singapore	White rice cooked with oil	68	4.3	194 g cooked white rice 30 g ground nut oil	50	12 Healthy participants	Glucose/2 h
11	^22^	Singapore	White rice served with chicken breast without skin	73	4.1	194 g cooked white rice 100 g chicken breast	50	12 Healthy participants	Glucose/2 h
12	^22^	Singapore	White rice served with leaf vegetables	82	5.8	194 g cooked rice 120 g vegetables	50	12 Healthy participants	Glucose/2 h
13	^22^	Singapore	White rice cooked with oil, served with chicken breast and leafy vegetables	50	4.0	194 g cooked rice 30 g ground nut oil 100 g chicken breast 120 g vegetables	50	12 Healthy participants	Glucose/2 h
14	^23^	Singapore	Jasmine rice	C: 91.2 M: 92.0 I: 90.2	C: 19.2 M: 20.0 I: 23.4	63.6 g raw (cooked with 130 ml water)	50	75 Healthy participants	Glucose/2 h
15	^23^	Singapore	Basmati rice	C: 55.7 M: 62.6 I: 59.5	C: 13.3 M: 13.1 I: 18.1	66.5 g raw (cooked with 170 ml water)	50	75 Healthy participants	Glucose/2 h
16	^24^	Singapore	Ice green tea	50	5	833 (ml)	50	13 Healthy participants	Glucose/2 h
17	^24^	Singapore	Ice lemon tea	74	7	500 (ml)	50	14 Healthy participants	Glucose/2 h
18	^24^	Singapore	Barley drink	62	6	658 (ml)	50	11 Healthy participants	Glucose/2 h
19	^24^	Singapore	Chinese carrot cake	77	8	316.9	50	10 Healthy participants	Glucose/2 h
20	^24^	Singapore	Beehoon	35	3	61.9	50	11 Healthy participants	Glucose/2 h
21	^24^	Singapore	Chinese yam cake	86	11	391.5	50	10 Healthy participants	Glucose/2 h
22	^24^	Singapore	Pandan waffle	46	6	148.3	50	11 Healthy participants	Glucose/2 h
23	^24^	Singapore	Chee cheong fun	81	7	277.4	50	10 Healthy participants	Glucose/2 h
24	^24^	Singapore	Lo mai gai	106	12	176.6	50	12 Healthy participants	Glucose/2 h
25	^24^	Singapore	Pink rice cake	97	12	184.8	50	11 Healthy participants	Glucose/2 h
26	^24^	Singapore	Curry puff	41	4	129.6	50	11 Healthy participants	Glucose/2 h
27	^24^	Singapore	Char siew (pork) pau	66	7	154.7	50	10 Healthy participants	Glucose/2 h
28	^24^	Singapore	Youtiao	55	4	109.6	50	11 Healthy participants	Glucose/2 h
29	^24^	Singapore	Kaya butter toast	49	4	108.7	50	11 Healthy participants	Glucose/2 h
30	^24^	Singapore	Nasi lemak	66	5	179.2	50	12 Healthy participants	Glucose/2 h
31	^25^	Singapore	White bread—Gardenia Brand	83	8.8	91.4	50	10 Healthy participants	Glucose/2 h
32	^25^	Singapore	White bread—Gardenia brand with essence of chicken (Cerebos Pacific Ltd., Singapore, Singapore)	56.9	7.5	91.4	50	10 Healthy participants	Glucose/2 h
33	^26^	Singapore	Wheat flour muffin	74.4	8.1	126.1	50	12 Healthy participants	Glucose/2 h
34	^26^	Singapore	Rice flour muffin	79.1	6.3	119.4	50	12 Healthy participants	Glucose/2 h
35	^26^	Singapore	Corn flour muffin	74.4	5.4	136.9	50	12 Healthy participants	Glucose/2 h
36	^26^	Singapore	Oat flour muffin	53.6	4.8	146.8	50	12 Healthy participants	Glucose/2 h
37	^26^	Singapore	Barley flour muffin	55.4	4.6	139.7	50	12 Healthy participants	Glucose/2 h
38	^27^	Singapore	White bread + soy milk	77.2	7.1	58 g White bread 322 ml Soy milk	50	12 Healthy participants	Glucose/2 h
39	^27^	Singapore	White bread + dairy milk	74.3	6.7	58 g White bread 322 ml Dairy milk	50	12 Healthy participants	Glucose/2 h
40	^28^	Singapore	Guava bites	28	7	NA	25	10 Healthy participants	Glucose/2 h
41	^28^	Singapore	Guava puree	45	6	NA	25	10 Healthy participants	Glucose/2 h
42	^28^	Singapore	Papaya bites	38	4	NA	25	10 Healthy participants	Glucose/2 h
43	^28^	Singapore	Papaya puree	40	6	NA	25	10 Healthy participants	Glucose/2 h
44	^29^	Singapore	Chinese steamed white bun	58	3	88	50	19 Healthy participants	Glucose/2 h
45	^29^	Singapore	White bun filled with red bean paste	58	4	106	50	19 Healthy participants	Glucose/2 h
46	^29^	Singapore	Rice idli	85	4	162	50	19 healthy participants	Glucose/2 h
47	^29^	Singapore	Rice dosa	76	5	193	50	19 Healthy participants	Glucose/2 h
48	^29^	Singapore	Upma	71	6	310	50	19 Healthy participants	Glucose/2 h
49	^29^	Singapore	Whole-grain biscuit	54	5	82	50	19 Healthy participants	Glucose/2 h
50	^29^	Singapore	Whole-grain biscuit filled with peanut butter	44	3	102	50	19 Healthy participants	Glucose/2 h
51	^29^	Singapore	Whole-grain oat muesli	55	4	84	50	19 Healthy participants	Glucose/2 h
52	^29^	Singapore	Whole-grain oat protein granola	51	4	87	50	19 Healthy participants	Glucose/2 h
53	^29^	Singapore	Whole-grain protein cereal	49	3	99	50	19 Healthy participants	Glucose/2 h
54	^30^	Singapore	White bread + rice bran soy milk (RBS)	83.1	7.7	89.5 bread + 195 (ml) RBS	50	17 Healthy participants	White bread/2 h
55	^30^	Singapore	White bread + sugar-free soy milk (SFS)	77.5	10.1	91.4 bread + 195 (ml) SFS	50	17 Healthy participants	White bread/2 h
56	^30^	Singapore	White bread	100	NA	95.8 bread + 195 (ml) water	50	17 Healthy participants	White Bread/2 h
57	^31^	Singapore	Basmati rice (Dreamrice™, Singapore)	55	15 (SD)	66.5 g (with 170 ml water)	50	75 Healthy participants	Glucose/2 h
58	^31^	Singapore	Jasmine rice (Double FP Thai Hom Mali premium quality fragrant rice, Thailand)	91	21 (SD)	63.6 g (with 130 ml water)	50	75 Healthy participants	Glucose/2 h
59	^32^	Malaysia	Multi-grains bread	56	6.2	57.9	25	12 Healthy participants	Glucose/2 h
60	^32^	Malaysia	Wholemeal bread with oatmeal	67	6.9	56.3	25	12 Healthy participants	Glucose/2 h
61	^32^	Malaysia	Wholemeal bread	85	5.9	89.0	25	12 Healthy participants	Glucose/2 h
62	^32^	Malaysia	White bread	83	6.5	51.9	25	12 Healthy participants	Glucose/2 h
63	^33^	Malaysia	Banana (*Musa paradisiaca*)	55	12	211	50	12 Healthy participants	White bread/2 h
64	^33^	Malaysia	Sweet potato (*Ipomoea batatas*)	77	12	162	50	12 Healthy participants	White bread/2 h
65	^33^	Malaysia	Rice noodles/kuay teow (*Oryza sativa*)	85	15	157	50	12 Healthy participants	White bread/2 h
66	^33^	Malaysia	White rice (*Oryza Sativ*a)	90	12	64	50	12 Healthy participants	White bread/2 h
67	^34^	Malaysia	Watermelon (*Citrulius vulgaris*—red variety)	55	3	893	50	10 Healthy participants	Glucose/2 h
68	^35^	Malaysia	Brown rice	51	8	NA	50	10 Healthy participants	Glucose/2 h
69	^35^	Malaysia	Polished brown rice	86	14	NA	50	10 Healthy participants	Glucose/2 h
70	^35^	Malaysia	White rice	79	14	NA	50	10 Healthy participants	Glucose/2 h
71	^36^	Malaysia	Rice	48	6	32	25	10 T2DM participants	Glucose/2 h
72	^36^	Malaysia	Lacy panckaes	49	6	35	25	10 T2DM participants	Glucose/2 h
73	^36^	Malaysia	Flatbread	71	2	54	25	10 T2DM participants	Glucose/2 h
74	^36^	Malaysia	Noodles	60	6	67	25	10 T2DM participants	Glucose/2 h
75	^36^	Malaysia	Coconut milk rice	49	7	NA	33	10 T2DM participants	Glucose/2 h
76	^36^	Malaysia	Lacy pancake with chicken curry	81	10	NA	32	10 T2DM participants	Glucose/2 h
77	^36^	Malaysia	Flatbread with dhal curry	69	7	NA	33	10 T2DM participants	Glucose/2 h
78	^36^	Malaysia	Fried noodles with chicken and prawns	55	8	NA	28	10 T2DM participants	Glucose/2 h
79	^37^	Malaysia	Chiffon cake	60	6	~122	25	11 Healthy participants	Glucose/2 h
80	^37^	Malaysia	Chiffon cake with 10% wheat flour replaced by young corn ear	49	4	~116	25	11 Healthy participants	Glucose/2 h
81	^38^	Malaysia	Bario celum rice	60.9	7.2	50	50	12 Healthy participants	Glucose/2 h
82	^38^	Malaysia	Bario tuan rice	62.2	8.9	50	50	12 Healthy participants	Glucose/2 h
83	^38^	Malaysia	Adan halus	72.1	9.5	50	50	12 Healthy participants	Glucose/2 h
84	^38^	Malaysia	Beras merah (red rice)	78.3	9.9	50	50	12 Healthy participants	Glucose/2 h
85	^39^	Malaysia	White rice (5% broken)	87.3	14.4	90.85	25	11 Healthy participants	Glucose/2 h
86	^39^	Malaysia	Fragrant white rice	124.2	16.4	102.87	25	11 Healthy participants	Glucose/2 h
87	^40^	Malaysia	Biscuit	61	13	37	25	11 Healthy participants	Glucose/2 h
88	^40^	Malaysia	Biscuit with 10% cornllete powder	46	11	39	25	11 Healthy participants	Glucose/2 h
89	^40^	Malaysia	Muffin	58	6	49	25	11 Healthy participants	Glucose/2 h
90	^40^	Malaysia	Muffin with 10% cornllete powder	57	9	63	25	11 Healthy participants	Glucose/2 h
91	^41^	Malaysia	Flatbread	63	4	71.4	50	10 Healthy participants	Glucose/2 h
92	^41^	Malaysia	Flatbread with 10% fenugreek	43	5	72	50	10 Healthy participants	Glucose/2 h
93	^41^	Malaysia	Bun	82	5	74	50	10 Healthy participants	Glucose/2 h
94	^41^	Malaysia	Bun with 10% fenugreek	138	51	91.1	50	10 Healthy participants	Glucose/2 h
95	^42^	Malaysia	Thai red	55	8.6	174.2	50	12 Healthy participants	Glucose/2 h
96	^42^	Malaysia	Basmati	50	5.8	188.3	50	12 Healthy participants	Glucose/2 h
97	^42^	Malaysia	Jasmine	78.7	11.6	180.3	50	12 Healthy participants	Glucose/2 h
98	^43^	Malaysia	Control biscuits without *Pleurotus sajor-caju* powder	57.2	4.8	76	25	11 Healthy participants	Glucose/2 h
99	^43^	Malaysia	Biscuits made with 4% *Pleurotus sajor-caju* powder	52	6.2	81	25	11 Healthy participants	Glucose/2 h
100	^43^	Malaysia	Biscuits made with 8% *Pleurotus sajor-caju* powder	49	6.5	85	25	11 Healthy participants	Glucose/2 h
101	^43^	Malaysia	Biscuits made with 12% *Pleurotus sajor-caju* powder	47.4	4.4	88	25	11 Healthy participants	Glucose/2 h
102	^44^	Malaysia	Fragrant white rice (Super fragrant AAA, Thailand)	67	7	77	25	11 Healthy participants	Glucose/2 h
103	^44^	Malaysia	Red rice (Jasmine Nutri Rice, Thailand)	68	8	84	25	11 Healthy participants	Glucose/2 h
104	^44^	Malaysia	Parboiled rice (Faiza Basmati, Malaysia	61	8	110	25	11 Healthy participants	Glucose/2 h
105	^44^	Malaysia	Fried fragrant white rice (Super fragrant AAA, Thailand),	50	7	129	25	11 Healthy participants	Glucose/2 h
106	^44^	Malaysia	Fried red rice (Jasmine Nutri Rice, Thailand)	41	4	139	25	11 Healthy participants	Glucose/2 h
107	^44^	Malaysia	Fried parboiled rice (Faiza Basmati, Malaysia	41	4	157	25	11 Healthy participants	Glucose/2 h
108	^45^	Malaysia	Red-fleshed seedless watermelon	51	2	236	25	14 Healthy participants	Glucose/2 h
109	^45^	Malaysia	Red-fleshed seeded watermelon	48	1	239	25	14 Healthy participants	Glucose/2 h
110	^45^	Malaysia	Yellow-fleshed watermelon	47	2	233	25	14 Healthy participants	Glucose/2 h
111	^45^	Malaysia	Red-fleshed seedless watermelon juice	51	1	236	25	14 Healthy participants	Glucose/2 h
112	^46^	Malaysia	Fried mihun—Terengganu	45.40	7.43	149	50	10 Healthy participants	Glucose/2 h
113	^46^	Malaysia	Fried kuay teow—Terengganu	79.50	9.34	178	50	10 Healthy participants	Glucose/2 h
114	^46^	Malaysia	Kuih (apam ayu)	90.56	12.0	106.2	50	10 Healthy participants	Glucose/2 h
115	^46^	Malaysia	Fish snacks (boiled keropok lekor) + chilli sauce (23 g)	79	9.5	129	50	10 Healthy participants	Glucose/2 h
116	^47^	Malaysia	Coconut sap sugar	42	4	27.7	25	10 Healthy participants	Glucose/2 h
117	^47^	Malaysia	Coconut sap syrup	39	4	32	25	10 Healthy participants	Glucose/2 h
118	^47^	Malaysia	Kaong sugar	43	3	26.4	25	10 Healthy participants	Glucose/2 h
119	^47^	Malaysia	Sorghum sugar	60	3	27.2	25	10 Healthy participants	Glucose/2 h
120	^47^	Malaysia	Buri sugar	57	3	29.3	25	10 Healthy participants	Glucose/2 h
121	^47^	Malaysia	Nipa sugar	58	2	28.8	25	10 Healthy participants	Glucose/2 h
122	^47^	Malaysia	Sugarcane granules	68	3	25.2	25	10 Healthy participants	Glucose/2 h
123	^47^	Malaysia	Muscovado sugar	69	3	26.3	25	10 Healthy participants	Glucose/2 h
124	^48^	Malaysia	Biscuit with durian (~19% w/w)	63.8	NA	6.0	50	10 Healthy participants	Glucose/2 h
125	^48^	Malaysia	Biscuit with oats (~9% w/w)	71.8	NA	5.5	50	10 Healthy participants	Glucose/2 h
126	^48^	Malaysia	Biscuit with durian (~17% w/w) and oats (~8% w/w)	59.4	NA	6.0	50	10 Healthy participants	Glucose/2 h
127	^49^	Malaysia	Beta-glucan drink (oat beta-1,3/1,4 glucan, Zhuhai City, China) with 80% purity	117	98	250 ml	50	10 Healthy participants	Glucose/2 h
128	^49^	Malaysia	Whey protein drink (Mesotropin Platinum Hydro Whey, Terengganu, Malaysia)	124	98	250 ml	50	10 Healthy participants	Glucose/2 h
129	^49^	Malaysia	Whey protein beta-glucan drink (4 g of beta-glucan per and 5 g whey protein per 250 ml)	114	97	250 ml	50	10 Healthy participants	Glucose/2 h
130	^50^	Thailand	Thailand Chiang brown rice—pressure cooked	58	8	92.2	25	10 Healthy participants	Glucose/2 h
131	^50^	Thailand	Thailand Sungyod brown rice—pressure cooked	81	9	91.3	25	10 Healthy participants	Glucose/2 h
132	^50^	Thailand	Thailand Lepnok brown rice—pressure cooked	59	6	90.5	25	10 Healthy participants	Glucose/2 h
133	^50^	Thailand	Malaysian Long grain specialty brown rice-1 (LS1)—pressure cooked	73	11	86.9	25	10 Healthy participants	Glucose/2 h
134	^50^	Thailand	Malaysian Long grain specialty brown rice-2 (LS2)—pressure cooked	65	6	87.9	25	10 Healthy participants	Glucose/2 h
135	^50^	Thailand	Thailand Chiang brown rice—cooked in rice cooker	65	7	89.8	25	10 Healthy participants	Glucose/2 h
136	^50^	Thailand	Thailand Sungyod brown rice—cooked in rice cooker	72	10	90.5	25	10 Healthy participants	Glucose/2 h
137	^50^	Thailand	Thailand Lepnok brown rice—cooked in rice cooker	62	10	89.4	25	10 Healthy participants	Glucose/2 h
138	^50^	Thailand	Malaysian Long grain specialty brown rice-1 (LS1)—cooked in rice cooker	64	6	88.0	25	10 Healthy participants	Glucose/2 h
139	^50^	Thailand	Malaysian Long grain specialty brown rice-2 (LS2)—cooked in rice cooker	72	7	85.9	25	10 Healthy participants	Glucose/2 h
140	^51^	Thailand	Big rice noodles from mixed sago palm flour (*Metroxylon* spp.) and Chiang rice flour	63.1	9.8	176 (fresh wet basis)	50	12 Healthy participants	Glucose/2 h
141	^51^	Thailand	Small rice noodles from mixed sago palm flour (*Metroxylon* spp.) and Chiang rice flour 60; 40	53.6	8.3	61 g (fresh wet basis)	50	12 Healthy participants	Glucose/2 h
142	^52^	Thailand	Wheat bread	77.8	4.6	128.1	50	16 Healthy participants	Glucose/2 h
143	^52^	Thailand	Riceberry rice bread	69.3	4.4	128.2	50	16 Healthy participants	Glucose/2 h
144	^52^	Thailand	Hom mali bread	130.6	7.9	115.7	50	16 Healthy participants	Glucose/2 h
145	^53^	Thailand	Parboiled brown rice (Leuang Awn)	50.10	5.37	167	50	12 Healthy participants	Glucose/2 h
146	^53^	Thailand	Germinated parboiled brown rice (Leuang Awn)	60.58	6.48	176	50	12 Healthy participants	Glucose/2 h
147	^53^	Thailand	Brown rice (Leuang Awn)	66.21	7.78	176	50	12 Healthy participants	Glucose/2 h
148	^53^	Thailand	Polished rice (Leuang Awn)	83.10	5.10	187	50	12 Healthy participants	Glucose/2 h
149	^54^	Indonesia	Steamed white rice	80	NA	NA	50	11 Healthy participants	White bread/2 h
150	^54^	Indonesia	Sukun (*Artocarpus communis* Forst)	90	NA	NA	50	11 Healthy participants	White bread/2 h
151	^54^	Indonesia	Banana (*Musa paradisiaca* fa. Typical), Pisang kepok	92	NA	NA	50	11 Healthy participants	White bread/2 h
152	^54^	Indonesia	Cassava	78	NA	NA	50	11 Healthy participants	White bread/2 h
153	^54^	Indonesia	Ubi/uwi (*Dioscorea alata* Linn.)	73	NA	NA	50	11 Healthy participants	White bread/2 h
154	^54^	Indonesia	Sorghum	160	NA	NA	50	11 Healthy participants	White bread/2 h
155	^55^	Indonesia	Taro (*Xanthosorna violaceum* Schott)	95	NA	128	50	10 Healthy participants	White bread/2 h
156	^55^	Indonesia	Yam (*Dioscorea aculeata* Linn.)	90	NA	186	50	10 Healthy participants	White bread/2 h
157	^55^	Indonesia	Edible canna (*Canna edulis* Ker)	105	NA	224	50	10 Healthy participants	White bread/2 h
158	^55^	Indonesia	Arrowroot (*Maranta arundinacea* Linn.	14	NA	300	25	10 Healthy participants	White bread/2 h
159	^55^	Indonesia	Sweet potato (*Ipomoea balatas* Poir)	179	NA	212	50	10 Healthy participants	White bread/2 h
160	^56^	Indonesia	Red bean (*Vigna umbellata*)	26	NA	84	25	11 Healthy participants	White bread/2 h
161	^56^	Indonesia	Mung bean (*Phaseolus aureus*)	76	NA	95	25	11 Healthy participants	White bread/2 h
162	^56^	Indonesia	Cowpea (*Vigna sinensis* Endl.)	35	NA	130	25	11 Healthy participants	White bread/2 h
163	^56^	Indonesia	Pigeon pea (*Cajanus cajan* Millspaugh)	51	NA	106	25	11 Healthy participants	White bread/2 h
164	^56^	Indonesia	Edible podded peas (*Pisum sativum* Linn.)	30	NA	178	25	11 Healthy participants	White bread/2 h
165	^56^	Indonesia	Soybean (*Glycine max* Merr.)	31	NA	138	25	11 Healthy participants	White bread/2 h
166	^57^	Indonesia	Kacang panjang/snap bean (*Phaseolus vulgaris*)	86	NA	146.39	25	10 Healthy participants	White bread/2 h
167	^57^	Indonesia	Buncis/yardlong Bean (*Vigna sesquipedalis*)	43	NA	105.28	25	10 Healthy participants	White bread/2 h
168	^58^	Indonesia	Arrowroot oyek	41	NA	65.95	50	10 Healthy participants	Glucose/2 h
169	^58^	Indonesia	Suweg oyek	42	NA	64.61	50	10 Healthy participants	Glucose/2 h
170	^58^	Indonesia	Cassava oyek	30	NA	61.71	50	10 Healthy participants	Glucose/2 h
171	^58^	Indonesia	Arrowroot tiwul (Garut)	40	NA	68.58	50	10 Healthy participants	Glucose/2 h
172	^58^	Indonesia	Suweg tiwul	40	NA	67.72	50	10 Healthy participants	Glucose/2 h
173	^58^	Indonesia	Cassava tiwul (Singkong)	29	NA	60.46	50	10 Healthy participants	Glucose/2 h
174	^59^	Indonesia	Standard cookies	67	NA	73	50	10 Healthy participants	Glucose/2 h
175	^59^	Indonesia	Cookies with rice bran	31	NA	87	50	10 Healthy participants	Glucose/2 h
176	^59^	Indonesia	Standard donut	72	NA	120	50	10 Healthy participants	Glucose/2 h
177	^59^	Indonesia	Donut made with rice bran	39	NA	119	50	10 Healthy participants	Glucose/2 h
178	^60^	Indonesia	Brown rice	97.28	NA	123	50	21 Healthy participants	White bread/2 h
179	^60^	Indonesia	White rice (IR 64)	99.26	NA	113	50	21 Healthy participants	White bread/2 h
180	^61^	Indonesia	Bread made from 400 g flour containing 50% w/w annealed purple yam flour	93.19	NA	95	50	10 Healthy participants	White bread/2 h
181	^62^	Indonesia	Boiled GEMBILI (*Dioscorea esculenta*)	85.56	NA	114.7	25	10 Healthy participants	Glucose/2 h
182	^62^	Indonesia	Steamed GEMBILI (*Dioscorea esculenta*)	87.56	NA	86.2	25	10 Healthy participants	Glucose/2 h
183	^62^	Indonesia	Fried GEMBILI (*Dioscorea esculenta*)	83.61	NA	76.2	25	10 Healthy participants	Glucose/2 h
184	^63^	Indonesia	Snack bar—red sweet potato	23.56	NA	90.91	50	10 Healthy participants	Glucose/2 h
185	^63^	Indonesia	Snack bar—yellow sweet potato	41.08	NA	78.12	50	10 Healthy participants	Glucose/2 h
186	^63^	Indonesia	Snack bar—purple sweet potato	21.54	NA	86.21	50	10 Healthy participants	Glucose/2 h
187	^64^	Indonesia	Wheat flour noodles	69.49	1.37	NA	50	10 Healthy participants	Glucose/2 h
188	^64^	Indonesia	Wheat flour noodle with 20% of wheat flour replaced with whole-wheat flour (dewata variety)	66.23	6.14	NA	50	10 Healthy participants	Glucose/2 h
189	^64^	Indonesia	Wheat flour cookies (with 0% whole-wheat flour)	52.11	2.07	NA	50	10 Healthy participants	Glucose/2 h
190	^64^	Indonesia	Wheat flour cookies with 20% of wheat flour replaced with whole-wheat flour (dewata variety)	49.94	1.90	NA	50	10 Healthy participants	Glucose/2 h
191	^65^	Indonesia	Standard wheat biscuit (with agar-agar and Diabetasol sweetener)	52.11	NA	NA	NA	10 Healthy participants	Glucose/2 h
192	^65^	Indonesia	Wheat biscuit substituted with 20% whole-wheat flour	49.94	NA	NA	NA	10 Healthy participants	Glucose/2 h
193	^66^	Indonesia	Analogue rice (80% mocaf:20% corn flour)	46.06	4.95	77.34	50	10 Healthy participants	White bread/2 h
194	^66^	Indonesia	Analogue rice (80% mocaf:20% sweet potato flour)	44.01	3.79	70.58	50	10 Healthy participants	White bread/2 h
195	^66^	Indonesia	Analogue rice (80% mocaf:20% carrot)	42.03	5.59	85.35	50	10 Healthy participants	White bread/2 h
196	^67^	Indonesia	Arrowroot snack bar with 30% kidney beans	25	NA	42	50	10 Healthy participants	Glucose/2 h
197	^68^	Indonesia	Optimum rice analogue formulation made from corn, sago, soybean and rice brans	54	NA	NA	50	10 Healthy participants	Glucose/2 h
198	^69^	Indonesia	Gayam seed (*Inocarfus fagifer* Forst. Gayam flour without pre-gelatinisation	74	NA	90.40	50	10 Healthy participants	Glucose/2 h
199	^69^	Indonesia	Pre-geletinised Gayam flour boiled for 15 min	75	NA	79.91	50	10 Healthy participants	Glucose/2 h
200	^69^	Indonesia	Pre-geletinised Gayam flour boiled for 30 min	61	NA	81.71	50	10 Healthy participants	Glucose/2 h
201	^69^	Indonesia	Pre-geletinised Gayam flour boiled for 45 min	57	NA	84.43	50	10 Healthy participants	Glucose/2 h
202	^70^	Indonesia	Corn-based rice analogues with 20% cassava starch	34.79	2.11 (SD)	NA	50	10 Healthy participants	Glucose/2 h
203	^70^	Indonesia	Corn-based rice analogues with 30% cassava starch	37.47	2.16 (SD)	NA	50	10 Healthy participants	Glucose/2 h
204	^70^	Indonesia	Corn-based rice analogues with 40% cassava starch	40.77	2.12 (SD)	NA	50	10 Healthy participants	Glucose/2 h
205	^71^	Indonesia	Millet and bean cookie bar (15% foxtail millet, 15% arrowroot flour and 30% of kidney beans)	37.6	NA	85	25	12 Healthy participants	Glucose/2 h
206	^72^	Indonesia	Arenga (*Arenga pinata*) starch cake	77.72	9.57	53.44	50	12 Healthy participants	Glucose/2 h
207	^72^	Indonesia	Arenga (*Arenga pinata*) starch cake with 4% guava extract	51.84	6.34	53.44	50	12 Healthy participants	Glucose/2 h
208	^72^	Indonesia	Arenga (*Arenga pinata*) starch cookie	47.31	6.22	72	50	12 Healthy participants	Glucose/2 h
209	^72^	Indonesia	Arenga (*Arenga pinata*) starch cookie with 4% guava extract	46.2	7.39	72	50	12 Healthy participants	Glucose/2 h
210	^73^	Indonesia	Steamed brownies made with wheat and sweet potato flour (1:1 ratio)	53.76	NA	140	50	10 Healthy participants	White bread/2 h
211	^74^	Indonesia	Fried noodle snack made with flour, yellow sweet potatoes (*Ipomoea batatas*) and pumpkin (*Cucurbita moschata*) flour	30.18	NA	35	25	10 Healthy participants	Glucose/2 h
212	^75^	Indonesia	Pumpkin flour chips	51	NA	88	50	10 Healthy participants	Glucose/2 h
213	^75^	Indonesia	Pumpkin flour chips with 15% bran flour substitution	87	NA	88	50	10 Healthy participants	Glucose/2 h
214	^76^	Indonesia	SIKKATO (Sinonggi)	78.42	NA	59.32	50	10 Healthy participants	White bread/2 h
215	^76^	Indonesia	SIKKATO (Kasuami)	90.36	NA	72.11	50	10 Healthy participants	White bread/2 h
216	^76^	Indonesia	SIKKATO (Kambuse)	72.04	NA	71.29	50	10 Healthy participants	White bread/2 h
217	^76^	Indonesia	SIKKATO (Kabuto)	84.54	NA	136.84	50	10 Healthy participants	White bread/2 h
218	^77^	Indonesia	Pekawai (*Durio kutejensis*; *Durian* species) chips	12	NA	NA	50	10 Healthy participants	Glucose/2 h
219	^78^	Indonesia	Coleus tuberosus crackers	40.88	6.42	NA	50	10 Healthy participants	Glucose/2 h
220	^78^	Indonesia	Wheat crackers	78.06	5.36	NA	50	10 Healthy participants	Glucose/2 h
221	^79^	Indonesia	Cookies made with brown rice flour (*Oryza nivara*) and winged bean’s (*Psophocarpus tetragonolobus* L.) seed flour	17.39	NA	146	50	10 Healthy participants	Glucose/2 h
222	^79^	Indonesia	Standard cookies	36.82	NA	92	50	10 Healthy participants	Glucose/2 h
223	^80^	Indonesia	Chromium-fortified parboiled rice (Cr-PR) coated with cinammon extracts	29	NA	NA	50	18 Healthy participants	Glucose/2 h
224	^81^	Indonesia	White sweet potato pudding (with agar-agar and Diabetasol sweetener)	37.75	NA	200.24	50	10 Healthy participants	Glucose/2 h
225	^81^	Indonesia	White sweet potato pudding with addition of red dragon fruit 25% (with agar-agar and Diabetasol sweetener)	33.81	NA	233.32	50	10 Healthy participants	Glucose/2 h
226	^81^	Indonesia	White sweet potato pudding with addition of red dragon fruit 50%, (with agar-agar and Diabetasol sweetener)	32.81	NA	286.20	50	10 Healthy participants	Glucose/2 h
227	^81^	Indonesia	White sweet potato pudding with addition of red dragon fruit 75%,	29.54	NA	349.90	50	10 Healthy participants	Glucose/2 h
228	^82^	Indonesia	Mocaf-black rice flakes with black soybean flour	50.19	21.57	63.8	50	10 Healthy participants	Glucose/2 h
229	^82^	Indonesia	Mocaf-black rice flakes added with jack bean flour	52.59	22.93	57.4	50	10 Healthy participants	Glucose/2 h
230	^83^	Indonesia	Honey tikung	35	NA	69	50	10 Healthy participants	Glucose/2 h
231	^83^	Indonesia	Honey kelulut	39	NA	73	50	10 Healthy participants	Glucose/2 h
232	^84^	Indonesia	Snack bar made from sagu flour, tempe and beras hitam	44	23.75 (SD)	92 (2:1 ratio of sago starch and tempe)	50	10 Healthy participants	Glucose/2 h
233	^84^	Indonesia	Snack bar made from sagu flour, tempe and beras hitam	46	18.42 (SD)	108 (1.5:1 ratio of sago starch and tempe)	50	10 Healthy participants	Glucose/2 h
234	^84^	Indonesia	Snack bar made from sagu flour, tempe and beras hitam	40	13.62 (SD)	129 (1:1 ratio of sago starch and tempe)	50	10 Healthy participants	Glucose/2 h
235	^85^	Indonesia	Tempeh gembus cookies (50% flour replacement)	47.01	11.08	85.18	50	28 Healthy participants	Glucose/2 h
236	^85^	Indonesia	Tempeh gembus cookies (50% flour replacement)	53.66	16.55	89.97	50	28 Healthy participants	Glucose/2 h
237	^85^	Indonesia	Cookies	68.67	12.28	95.45	50	28 Healthy participants	Glucose/2 h
238	^86^	Indonesia	Corn flour cake	85.02	11.21	54	50	12 Healthy participants	Glucose/2 h
239	^86^	Indonesia	Cake made from modified corn flour (corn starch soaked with 4% green tea extract)	74.96	10.48	54	50	12 Healthy participants	Glucose/2 h
240	^86^	Indonesia	Corn flour cookie	52.23	6.78	71	50	12 Healthy participants	Glucose/2 h
241	^86^	Indonesia	Cookie made from modified corn flour with green tea extract	58.25	8.33	71	50	12 Healthy participants	Glucose/2 h
242	^87^	Indonesia	Sorghum, oatmeal and honey snack bar coated with caramel syrup made with sorghum	44.73	8.83	60.80	50	12 Healthy participants	Glucose/2 h
243	^87^	Indonesia	Sorghum, oatmeal and honey snack bar coated with caramel sugarcane syrup	53.72	3.63	57.87	50	12 Healthy participants	Glucose/2 h
244	^87^	Indonesia	Sorghum, oatmeal and honey snack bar coated with glucose syrup	81.41	8.17	58.20	50	12 Healthy participants	Glucose/2 h
245	^88^	Philippines	Pan de sal + coconut flour	87.2	5.5	NA	50	10 Healthy participants	Glucose/2 h
246	^88^	Philippines	Pan de sal + coconut flour	96.6	6.1	NA	50	10 T2DM participants	Glucose/3 h
247	^88^	Philippines	Granola bar + coconut flour	65.1	4.9	NA	50	10 Healthy participants	Glucose/2 h
248	^88^	Philippines	Granola bar + coconut flour	71.6	4.7	NA	50	10 T2DM participants	Glucose/3 h
249	^88^	Philippines	Cinnamon bread + coconut flour	62.7	4.2	NA	50	10 Healthy participants	Glucose/2 h
250	^88^	Philippines	Cinnamon bread + coconut flour	71.4	4.9	NA	50	10 T2DM participants	Glucose/3 h
251	^88^	Philippines	Multigrain loaf + coconut flour	85.2	6.8	NA	50	10 Healthy participants	Glucose/2 h
252	^88^	Philippines	Multigrain loaf + coconut flour	92.5	5.9	NA	50	10 T2DM participants	Glucose/3 h
253	^88^	Philippines	Choco chip cookies + coconut flour	61.3	4.6	NA	50	10 Healthy participants	Glucose/2 h
254	^88^	Philippines	Choco chip cookies + coconut flour	71.4	7.3	NA	50	10 T2DM participants	Glucose/3 h
255	^88^	Philippines	Hotcake + coconut flour	65.0	3.3	NA	50	10 Healthy participants	Glucose/2 h
256	^88^	Philippines	Hotcake + coconut flour	72.3	5.8	NA	50	10 T2DM participants	Glucose/3 h
257	^88^	Philippines	Choco crinkles + coconut flour	61.3	5.4	NA	50	10 Healthy participants	Glucose/2 h
258	^88^	Philippines	Choco crinkles + coconut flour	77.0	4.4	NA	50	10 T2DM participants	Glucose/3 h
259	^88^	Philippines	European carrot cake + coconut flour	51.8	3.3	NA	50	10 Healthy participants	Glucose/2 h
260	^88^	Philippines	European carrot cake + coconut flour	55.0	3.7	NA	50	10 T2DM participants	Glucose/3 h
261	^88^	Philippines	Macaroons + coconut flour	45.7	3.0	NA	50	10 Healthy participants	Glucose/2 h
262	^88^	Philippines	Macaroons + coconut flour	46.6	3.7	NA	50	10 T2DM participants	Glucose/3 h
263	^88^	Philippines	Brownies + coconut flour	60.1	5.4	NA	50	10 Healthy participants	Glucose/2 h
264	^88^	Philippines	Brownies + coconut flour	61.3	5.6	NA	50	10 T2DM participants	Glucose/3 h
265	^89^	Philippines	White bread	93.3	8.9	NA	50	11 Healthy participants	Glucose/2 h
266	^89^	Philippines	Japonica rice	87.5	7.8	NA	50	11 Healthy participants	Glucose/2 h
267	^89^	Philippines	Japonica rice + 3 g sunfibre	67.5	6.0	NA	50	11 Healthy participants	Glucose/2 h
268	^89^	Philippines	Japonica rice + 5 g sunfibre	65.5	5.8	NA	50	11 Healthy participants	Glucose/2 h
269	^89^	Philippines	White bread + 5 g sunfibre (drink)	49.0	4.4	NA	50	11 Healthy participants	Glucose/2 h
270	^89^	Philippines	White bread + 10 g sunfibre (drink)	56.9	5.1	NA	50	11 Healthy participants	Glucose/2 h
271	^89^	Philippines	White bread + 10 g inulin (drink)	66.7	6.0	NA	50	11 Healthy participants	Glucose/2 h
272	^89^	Philippines	White bread + 10 g Indigestible dextrin (drink)	66.3	5.9	NA	50	11 Healthy participants	Glucose/2 h
273	^90^	Philippines	Biscuit 1 (Marie)	88	7	44	25	10 Healthy participants	Glucose/2 h
274	^90^	Philippines	Biscuit 2 (Mik Mik)	94	7	45	25	10 Healthy participants	Glucose/2 h
275	^90^	Philippines	Biscuit 2 + oat fibre	52	4	37	25	10 Healthy participants	Glucose/2 h
276	^90^	Philippines	Donut, sugar coated	70	5	109	50	10 Healthy participants	Glucose/2 h
277	^90^	Philippines	Mamon, ordinary	48	3	60	25	10 Healthy participants	Glucose/2 h
278	^90^	Philippines	Mamon, sugar-free	48	4	55	25	10 Healthy participants	Glucose/2 h
279	^90^	Philippines	Kutsinta	80	6	65	25	10 Healthy participants	Glucose/2 h
280	^90^	Philippines	Puto, white	90	6	50	25	10 Healthy participants	Glucose/2 h
281	^90^	Philippines	Bihon (noodles)	49	3	61	50	10 Healthy participants	Glucose/2 h
282	^90^	Philippines	Canton (noodles)	49	2	97	50	10 Healthy participants	Glucose/2 h
283	^90^	Philippines	Sotanghon (noodles)	60	3	59	50	10 Healthy participants	Glucose/2 h
284	^90^	Philippines	Misua (noodles)	46	4	71	50	10 Healthy participants	Glucose/2 h
285	^90^	Philippines	Miki (noodles)	47	3	99	50	10 Healthy participants	Glucose/2 h
286	^90^	Philippines	Potato	43	3	118	50	10 Healthy participants	Glucose/2 h
287	^90^	Philippines	Yacon (tuber)	34	3	244	25	10 healthy participants	Glucose/2 h
288	^90^	Philippines	Yacon juice	61	2	250 (ml)	30	10 Healthy participants	Glucose/2 h
289	^90^	Philippines	Cashew nuts	36	4	106	25	10 Healthy participants	Glucose/2 h
290	^90^	Philippines	Lima beans	16	2	64	50	10 Healthy participants	Glucose/2 h
291	^90^	Philippines	Sitaw (string beans)	23	1	200	10	10 Healthy participants	Glucose/2 h
292	^90^	Philippines	Banana, Lakatan	62	5	180	50	10 Healthy participants	Glucose/2 h
293	^90^	Philippines	Banana, Saba	53	4	161	50	10 Healthy participants	Glucose/2 h
294	^90^	Philippines	Grapes, seedless	46	3	267	50	10 Healthy participants	Glucose/2 h
295	^90^	Philippines	Pear, Chinese	29	3	243	25	10 Healthy participants	Glucose/2 h
296	^90^	Philippines	Cantalope (melon)	34	3	291	25	10 Healthy participants	Glucose/2 h
297	^90^	Philippines	Watermelon	48	4	373	25	10 Healthy participants	Glucose/2 h
298	^90^	Philippines	Jackfruit	41	3	114	25	10 Healthy participants	Glucose/2 h
299	^90^	Philippines	Mango, carabao, ripe	46	4	176	25	10 Healthy participants	Glucose/2 h
300	^90^	Philippines	Papaya	45	3	232	25	10 Healthy participants	Glucose/2 h
301	^90^	Philippines	Apple, red	42	3	181	25	10 Healthy participants	Glucose/2 h
302	^90^	Philippines	Pineapple	56	3	215	25	10 Healthy participants	Glucose/2 h
303	^90^	Philippines	Guava, white	19	2	233	25	10 Healthy participants	Glucose/2 h
304	^90^	Philippines	Raisins	61	5	76	50	10 Healthy participants	Glucose/2 h
305	^90^	Philippines	Squash (veg)	44	5	234	15	10 Healthy participants	Glucose/2 h
306	^90^	Philippines	Carrot	35	2	211	15	10 Healthy participants	Glucose/2 h
307	^90^	Philippines	Sayote (veg)	27	2	286	10	10 Healthy participants	Glucose/2 h
308	^90^	Philippines	Togue (veg)	25	2	137	10	10 Healthy participants	Glucose/2 h
309	^90^	Philippines	Avocado	31	3	114	10	10 Healthy participants	Glucose/2 h
310	^90^	Philippines	Coconut sap sugar (PCA)	35	4	54	50	10 Healthy participants	Glucose/2 h
311	^90^	Philippines	Coconut sap sugar (e-Asia)	42	4	28	25	10 Healthy participants	Glucose/2 h
312	^90^	Philippines	Coconut sap syrup (e-Asia)	39	4	33	25	10 Healthy participants	Glucose/2 h
313	^91^	Japan	White rice − reference food (beihan) + dried sea algae	100	NA	147 g Rice + 1 g dried sea algae (shiso)	50.4	58 Healthy participants	White rice/2 h
314	^91^	Japan	Rice gruel (okayu)	99	38	659	50.2	10 Healthy participants	White rice/2 h
315	^91^	Japan	Rice cracker (osenbe)	111	44	25 pieces	50	10 Healthy participants	White rice/2 h
316	^91^	Japan	Low protein white rice (tei-tanpaku gohan)	86	28	125	50.4	10 Healthy participants	White rice/2 h
317	^91^	Japan	White rice and sated plum frout (umeboshi)	98	49	152	49.9	10 Healthy participants	White rice/2 h
318	^91^	Japan	White rice and curry	82	33	224	50.9	10 Healthy participants	White rice/2 h
319	^91^	Japan	White rice and pickled food (beihan, sunomono) (taken before rice)	73	29	173	49.8	11 Healthy participants	White rice/2 h
320	^91^	Japan	Butter rice	96	48	157	50	10 Healthy participants	White rice/2 h
321	^91^	Japan	White rice and yoghurt (taken before rice)	72	28	232	50.2	10 Healthy participants	White rice/2 h
322	^91^	Japan	White rice and yoghurt (taken after rice)	71	24	232	50.2	10 Healthy participants	White rice/2 h
323	^91^	Japan	White rice with curry and cheese	67	34	255	50.1	10 Healthy participants	White rice/2 h
324	^91^	Japan	White rice and fermented soybean (natto)	68	30	174	49.9	10 Healthy participants	White rice/2 h
325	^91^	Japan	Soybean paste soup (miso shiru) and rice	74	17	160	50.1	10 Healthy participants	White rice/2 h
326	^91^	Japan	Bread (International Standard Reference Food—white bread)	92	38	116	50.1	10 Healthy participants	White rice/2 h
327	^91^	Japan	Spaghetti	56	37	131	50.2	10 Healthy participants	White rice/2 h
328	^92^	Japan	White rice	75.9	6.6	161	49.3	19 Healthy participants	Glucose/2 h
329	^92^	Japan	Pre-germinated brown rice	56.9	2.9	185	50.6	19 Healthy participants	Glucose/2 h
330	^92^	Japan	Brown rice	61.5	4.7	178	49.4	19 Healthy participants	Glucose/2 h
331	^92^	Japan	1/3 Pre-germinated brown rice (mixture of pre-germinated brown rice to white rice)	67.4	2.9	169 (WR/PGBR ratio is 2:1)	49.7	13 Healthy participants	Glucose/2 h
332	^92^	Japan	2/3 Pre-germinated brown rice (mixture of pre-germinated brown rice to white rice)	63.7	5.3	177 (WR/PGBR ratio is 1:2)	50.2	13 Healthy participants	Glucose/2 h
333	^92^	Japan	White rice	74.6	6.2	161	49.3	13 Healthy participants	Glucose/2 h
334	^92^	Japan	Pre-germinated brown rice	54.4	5.1	185	50.6	13 Healthy participants	Glucose/2 h
335	^93^	Japan	Cake made from whole soy	22	6	114	50.5	20 Healthy participants	Glucose (50 g CHO)/4 h
336	^94^	Japan	Rice-1 (Sato-no-gohan)	71	25 (SD)	150	50	12 Healthy participants	Glucose/2 h
337	^94^	Japan	Rice-1 (Sato-no-gohan)	86	28 (SD)	150	50	12 Healthy participants	Glucose/3 h
338	^94^	Japan	Rice-2 (Nihonbare)	69	28 (SD)	135	50.1	12 Healthy participants	Glucose/2 h
339	^94^	Japan	Rice-2 (Nihonbare)	82	34 (SD)	135	50.1	12 Healthy participants	Glucose/3 h
340	^94^	Japan	Rice-3 (Hinohikari)	74	23 (SD)	142	50.1	12 Healthy participants	Glucose/2 h
341	^94^	Japan	Rice-3 (Hinohikari)	82	24 (SD)	142	50.1	12 Healthy participants	Glucose/3 h
342	^94^	Japan	Rice-4 (Koshihikari)	75	14 (SD)	142	50.1	12 Healthy participants	Glucose/2 h
343	^94^	Japan	Rice-4 (Koshihikari)	88	17 (SD)	142	50.1	12 Healthy participants	Glucose/3 h
344	^94^	Japan	Potato-1 (Nishiyutaka)	64	15 (SD)	284	50	12 Healthy participants	Glucose/2 h
345	^94^	Japan	Potato-1 (Nishiyutaka)	65	17 (SD)	284	50	12 Healthy participants	Glucose/3 h
346	^94^	Japan	Potato-2 (Ainoaka)	63	19 (SD)	284	50	12 Healthy participants	Glucose/2 h
347	^94^	Japan	Potato-2 (Ainoaka)	63	19 (SD)	284	50	12 Healthy participants	Glucose/3 h
348	^94^	Japan	Potato-3 (Dejima)	54	17 (SD)	284	50	12 Healthy participants	Glucose/2 h
349	^94^	Japan	Potato-3 (Dejima)	52	17 (SD)	284	50	12 Healthy participants	Glucose/3 h
350	^94^	Japan	Noodle-1 (Simabara-udon)	62	27 (SD)	170	50	12 Healthy participants	Glucose/2 h
351	^94^	Japan	Noodle-1 (Simabara-udon)	80	36 (SD)	170	50	12 Healthy participants	Glucose/3 h
352	^94^	Japan	Noodle-2 (Goto-udon)	38	15 (SD)	170	50	12 Healthy participants	Glucose/2 h
353	^94^	Japan	Noodle-2 (Goto-udon)	49	20 (SD)	170	50	12 Healthy participants	Glucose/3 h
354	^94^	Japan	Noodle-3 (Katokichi-udon)	55	7 (SD)	172	49.9	12 Healthy participants	Glucose/2 h
355	^94^	Japan	Noodle-3 (Katokichi-udon)	67	15 (SD)	172	49.9	12 Healthy participants	Glucose/3 h
356	^94^	Japan	White bread (Yamazaki)	58	25 (SD)	107	49.9	12 Healthy participants	Glucose/2 h
357	^94^	Japan	White bread (Yamazaki)	59	15 (SD)	107	49.9	12 Healthy participants	Glucose/3 h
358	^94^	Japan	Sponge cake (Castilla)	64	20 (SD)	80	49.9	12 Healthy participants	Glucose/2 h
359	^94^	Japan	Sponge cake (Castella)	65	19 (SD)	80	49.9	12 Healthy participants	Glucose/3 h
360	^95^	Japan	White rice	89	NA	NA	50	15 Healthy participants	Glucose/2 h
361	^95^	Japan	Long grain rice	60	NA	NA	50	15 Healthy participants	Glucose/2 h
362	^95^	Japan	Rice vermicelli (a)	55	NA	NA	50	15 Healthy participants	Glucose/2 h
363	^95^	Japan	Rice vermicelli (b)	50	NA	NA	50	15 Healthy participants	Glucose/2 h
364	^95^	Japan	Rice vermicelli (c)	35	NA	NA	50	15 Healthy participants	Glucose/2 h
365	^95^	Japan	Rice vermicelli (d)	59	NA	NA	50	15 Healthy participants	Glucose/2 h
366	^95^	Japan	Rice vermicelli (e)	60	NA	NA	50	15 Healthy participants	Glucose/2 h
367	^95^	Japan	Rice vermicelli (f)	62	NA	NA	50	15 Healthy participants	Glucose/2 h
368	^96^	Japan	Raw herb: corn salad	97.5	18.4 (SD)	20	50	11 Healthy participants	White rice/2 h
369	^96^	Japan	Herbal tea: lemon balm	99.6	22.2 (SD)	1	50	10 Healthy participants	White rice/2 h
370	^96^	Japan	Herbal tea: lemongrass	112.1	28.9 (SD)	1	50	10 Healthy participants	White rice/2 h
371	^96^	Japan	Herbal tea: rosemary	126.5	27.3 (SD)	0.6	50	10 Healthy participants	White rice/2 h
372	^96^	Japan	Herbal tea: spearmint	108.8	30 (SD)	0.5	50	10 Healthy participants	White rice/2 h
373	^96^	Japan	Herbal tea: thyme	106.1	22.6 (SD)	1	50	10 Healthy participants	White rice/2 h
374	^97^	Japan	Boiled Barleymax	24.3	2.5	204	50	11 Healthy participants	Glucose/2 h
375	^98^	Japan	Noodles made from dehulled yellow pea	50.4	31.6 (SD)	NA	NA	50	11 Healthy participants
376	^98^	Japan	Noodles made from dehulled yellow pea	40.3	25.3 (SD)	NA	50	11 Healthy participants	Glucose/2 h
377	^98^	Japan	Noodles made from unshelled yellow pea	68.8	12.4 (SD)	NA	50	11 Healthy participants	White rice/2 h
378	^98^	Japan	Noodles made from dehulled yellow pea	40.3	25.3 (SD)	NA	50	11 Healthy participants	Glucose/2 h
379	^99^	Korea	Apple	33.5	11.92 (SD)	100	50	13 Healthy participants	Glucose/2 h
380	^99^	Korea	Tangerine	50.4	15.16 (SD)	100	50	13 Healthy participants	Glucose/2 h
381	^99^	Korea	Pear	35.7	14.38 (SD)	100	50	13 Healthy participants	Glucose/2 h
382	^99^	Korea	Watermelon	53.5	18.07 (SD)	100	50	13 Healthy participants	Glucose/2 h
383	^99^	Korea	Persimmon	42.9	18.92 (SD)	100	50	13 Healthy participants	Glucose/2 h
384	^99^	Korea	Grapes	48.1	14.05 (SD)	100	50	13 Healthy participants	Glucose/2 h
385	^99^	Korea	Oriental melon	51.2	18.14 (SD)	100	50	13 Healthy participants	Glucose/2 h
386	^99^	Korea	Peach	56.5	14.17 (SD)	100	50	13 Healthy participants	Glucose/2 h
387	^100^	Korea	Rice gruel	92.5	8.8	447.7	50	10 Healthy participants	Glucose/2 h
388	^100^	Korea	Puffed rice grains	72.4	6.6	56.2	50	10 Healthy participants	Glucose/2 h
389	^100^	Korea	Rice cakes	80.7	8.5	93.8	50	10 Healthy participants	Glucose/2 h
390	^100^	Korea	Steamed glutinous rice	75.7	10.6	111.11	50	10 Healthy participants	Glucose/2 h
391	^100^	Korea	Rice balls	96.9	15.1	100	50	10 Healthy participants	Glucose/2 h
392	^100^	Korea	Barley powder	69.8	6.7	67.0	50	11 Healthy participants	Glucose/2 h
393	^100^	Korea	Fine noodles	49.0	7.0	65.8	50	13 Healthy participants	Glucose/2 h
394	^100^	Korea	Fresh wheat noodles	48.2	4.9	91.5	50	13 Healthy participants	Glucose/2 h
395	^100^	Korea	Hand-pulled dough	50.2	5.6	91.4	50	14 Healthy participants	Glucose/2 h
396	^100^	Korea	Spaghetti	55.3	6.5	72.5	50	11 Healthy participants	Glucose/2 h
397	^100^	Korea	Buckwheat noodles	59.6	13.3	70.2	50	13 Healthy participants	Glucose/2 h
398	^100^	Korea	Sweet potato starch vermicelli	60.0	11.6	56.8	50	11 Healthy participants	Glucose/2 h
399	^100^	Korea	Plainbread	70.7	11.4	116.6	50	10 Healthy participants	Glucose/2 h
400	^100^	Korea	Rye bread	64.9	18.4	109.4	50	10 Healthy participants	Glucose/2 h
401	^100^	Korea	Rice bread	73.4	7.6	116.6	50	11 Healthy participants	Glucose/2 h
402	^100^	Korea	Castella	59.9	13.3	114.2	50	10 Healthy participants	Glucose/2 h
403	^100^	Korea	Soft roll	56.2	11.1	103.5	50	10 Healthy participants	Glucose/2 h
404	^100^	Korea	Bagel	77.4	11.5	104.1	50	11 Healthy participants	Glucose/2 h
405	^100^	Korea	Wheat pancakes	57.0	9.7	102.8	50	14 Healthy participants	Glucose/2 h
406	^100^	Korea	Buckwheat pancakes	49.9	8.9	169.4	50	13 Healthy participants	Glucose/2 h
407	^100^	Korea	Cornflakes (Kellogg’s Inc., South Korea)	51.6	10.7	56.2	50	14 Healthy participants	Glucose/2 h
408	^100^	Korea	All-Bran (Kellogg’s Inc., South Korea)	51.4	11.1	57.5	50	11 Healthy participants	Glucose/2 h
409	^100^	Korea	Acorn jelly	71.7	16.0	361.2	50	12 Healthy participants	Glucose/2 h
410	^100^	Korea	Green bean jelly	55.1	8.9	443.2	50	14 Healthy participants	Glucose/2 h
411	^100^	Korea	Buckwheat jelly	65.7	11.8	318.5	50	13 Healthy participants	Glucose/2 h
412	^100^	Korea	Potato starch steamed	53.3	17.3	109.3	50	12 Healthy participants	Glucose/2 h
413	^100^	Korea	Baked sweet potatoes	90.9	9.6	160.3	50	10 Healthy participants	Glucose/2 h
414	^100^	Korea	Steamed chestnuts	57.8	6.3	134.8	50	13 Healthy participants	Glucose/2 h
415	^100^	Korea	Baked chestnuts	54.3	5.8	134.8	50	11 Healthy participants	Glucose/2 h
416	^100^	Korea	Steamed maize	73.4	9.9	170.1	50	11 Healthy participants	Glucose/2 h
417	^100^	Korea	Red bean gruel	38.5	7.3	247.9	50	10 Healthy participants	Glucose/2 h
418	^100^	Korea	Steamed sweet pumpkin	52.1	14.0	277.8	50	11 Healthy participants	Glucose/2 h
419	^101^	China	Cooked rice	83.2	3.1	NA	50	12 Healthy participants	Glucose/2 h
420	^101^	China	Brown rice (cooked)	87.0	5.0	NA	50	10 Healthy participants	Glucose/2 h
421	^101^	China	Sticky rice (cooked)	87.0	7.0	NA	50	10 Healthy participants	Glucose/2 h
422	^101^	China	Sticky rice (higher amylose)	50.0	6.0	NA	50	10 Healthy participants	Glucose/2 h
423	^101^	China	Rice porridge	69.4	18.5	NA	50	10 Healthy participants	Glucose/2 h
424	^101^	China	Instant rice (in hot water 3 min)	46.0	8.5	NA	50	10 Healthy participants	Glucose/2 h
425	^101^	China	Instant rice (cooked 6 min)	87.0	5.5	NA	50	10 Healthy participants	Glucose/2 h
426	^101^	China	Corn powder porridge	68.0	10.6	NA	50	10 Healthy participants	Glucose/2 h
427	^101^	China	Corn granule	51.8	9.2	NA	50	10 Healthy participants	Glucose/2 h
428	^101^	China	Sweet corn (cooked)	55.0	5.0	NA	50	10 Healthy participants	Glucose/2 h
429	^101^	China	Oat biscuit	55.0	2.5	NA	50	10 Healthy participants	Glucose/2 h
430	^101^	China	Wheat pancake	79.6	11.5	NA	50	10 Healthy participants	Glucose/2 h
431	^101^	China	Bread (refined wheat)	87.9	10.2	NA	50	10 Healthy participants	Glucose/2 h
432	^101^	China	Bread (whole wheat)	69.0	10.4	NA	50	10 Healthy participants	Glucose/2 h
433	^101^	China	Bread (whole wheat with dried fruit)	47.0	7.0	NA	50	10 Healthy participants	Glucose/2 h
434	^101^	China	Wheat noodle (dried)	46.0	5.8	NA	50	10 Healthy participants	Glucose/2 h
435	^101^	China	Dumpling (shallot + meat)	28.0	9.9	NA	50	10 Healthy participants	Glucose/2 h
436	^101^	China	Steamed stuffed bun (shallot + meat)	39.1	13.0	NA	50	10 Healthy participants	Glucose/2 h
437	^101^	China	Cake crisp	59.0	6.0	NA	50	10 Healthy participants	Glucose/2 h
438	^101^	China	Whole-wheat pancake	42.0	7.5	NA	50	10 Healthy participants	Glucose/2 h
439	^101^	China	WoTao (corn + wheat)	64.9	16.5	NA	50	10 Healthy participants	Glucose/2 h
440	^101^	China	Potato (cooked)	66.4	3.8	NA	50	10 healthy participants	Glucose/2 h
441	^101^	China	Potato (steam)	62.0	5.7	NA	50	10 Healthy participants	Glucose/2 h
442	^101^	China	Potato crisp (oil fry)	60.3	7.0	NA	50	10 Healthy participants	Glucose/2 h
443	^101^	China	Yam (steam)	51.0	12.0	NA	50	10 Healthy participants	Glucose/2 h
444	^101^	China	Yam (cooked)	54.0	5.5	NA	50	10 Healthy participants	Glucose/2 h
445	^101^	China	Potato mashed	73.0	9.2	NA	50	10 Healthy participants	Glucose/2 h
446	^102^	China	Resistant starch rice	48.4	21.8	NA	40	16 Healthy participants	Glucose/4 h
447	^102^	China	Wild-type rice	77.4	34.9	NA	40	16 Healthy participants	Glucose/4 h
448	^103^	China	MSB, millet steamed bread	89.6	8.8	100	50	10 Healthy participants	Glucose/2 h
449	^103^	China	MP-1, no. 1 millet pancake (75.0% millet flour and 25.0% extrusion flour)	83.0	9.6	141	50	10 Healthy participants	Glucose/2 h
450	^103^	China	MP-2, no. 2 millet pancake (without extrusion flour)	76.2	10.7	121	50	10 Healthy participants	Glucose/2 h
451	^103^	China	Cooked millet	64.4	8.5	169	50	10 Healthy participants	Glucose/2 h
452	^103^	China	Millet porridge	93.6	11.3	550	50	10 Healthy participants	Glucose/2 h
453	^104^	China	Majia pomelo	78.34	1.88	72.09 ± 1.08 g (fresh weight)	50	20 Healthy participants	Glucose/2 h
454	^104^	China	Majia pomelo	72.15	1.95	72.09 ± 1.08 g (fresh weight)	50	20 T2DM participants	Glucose/2 h
455	^105^	China	Rice	81	4	66.1	50	11 Healthy participants	Glucose and rice/4 h
456	^105^	China	Raisins	56	5	75.2	50	11 Healthy participants	Glucose and rice/4 h
457	^105^	China	Dried apples	43	4	76.8	50	11 Healthy participants	Glucose and rice/4 h
458	^105^	China	Dried jujubes	55	6	84.0	50	11 Healthy participants	Glucose and rice/4 h
459	^105^	China	Dried apricots	56	4	90.4	50	11 Healthy participants	Glucose and rice/4 h
460	^105^	China	Raisins + rice	77	8	37.6 (raisins) 33.1 (rice)	50	11 Healthy participants	Glucose and rice/4 h
461	^105^	China	Dried apples + rice	65	5	38.4 (dried apples) 33.1 (rice)	50	11 Healthy participants	Glucose and rice/4 h
462	^105^	China	Dried jujubes + rice	77	6	42.0 (dried jujubes) 33.1 (rice)	50	11 Healthy participants	Glucose and rice/4 h
463	^105^	China	Dried apricots + rice	75	7	45.2 (dried apricots) 33.1 (rice)	50	11 Healthy participants	Glucose and rice/4 h
464	^105^	China	Rice + almonds	70	4	66.1 (rice) 30 (almonds)	52	11 Healthy participants	Glucose and rice/4 h
465	^105^	China	Raisins + rice + almonds	54	2	37.6 (raisins) 33.1 (rice) 30 (almonds)	52	11 Healthy participants	Glucose and rice/4 h
466	^105^	China	Dried apples + rice + almonds	60	4	38.4 (dried apples) 33.1 (rice) 30 (almonds)	52	11 Healthy participants	Glucose and rice/4 h
467	^105^	China	Dried jujubes + rice + almonds	52	4	42.0 (dried jujubes) 33.1 (rice) 30 (almonds)	52	11 Healthy participants	Glucose and rice/4 h
468	^105^	China	Dried apricots + rice + almonds	64	4	45.2 (dried apricots) 3.1 (rice) 30 (almonds)	52	11 Healthy participants	Glucose and rice/4 h
469	^106^	China	Cooked rice + cooked pak choy	71	7	66.1 (raw rice) 300 (vegetables) 2.5 (sesame oil) 1.5 (salt)	52.9	16 Healthy participants	Glucose and rice/4 h
470	^106^	China	Cooked rice + homogenised raw pak choy	84	9	66.1 (raw rice) 300 (vegetables) 2.5 (sesame oil) 1.5 (salt)	52.9	16 Healthy participants	Glucose and rice/4 h
471	^106^	China	Cooked rice + homogenised cooked pak choy	91	10	66.1 (raw rice) 300 (vegetables) 2.5 (sesame oil) 1.5 (salt)	52.9	16 Healthy participants	Glucose and rice/4 h
472	^106^	China	Cooked rice + cooked cauliflower	73	7	66.1 (raw rice) 300 (vegetables) 2.5 (sesame oil) 1.5 (salt)	50.8	16 Healthy participants	Glucose and rice/4 h
473	^106^	China	Cooked rice + homogenised raw Cauliflower	83	10	66.1 (raw rice) 300 (vegetables) 2.5 (sesame oil) 1.5 (salt)	50.8	16 Healthy participants	Glucose and rice/4 h
474	^106^	China	Cooked rice + homogenised cooked Cauliflower	85	9	66.1 (raw rice) 300 (vegetables) 2.5 (sesame oil) 1.5 (salt)	50.8	16 Healthy participants	Glucose and rice/4 h
475	^106^	China	Cooked rice + cooked eggplant	67	8	66.1 (raw rice) 300 (vegetables) 2.5 (sesame oil) 1.5 (salt)	53.5	16 Healthy participants	Glucose and rice/4 h
476	^106^	China	Cooked rice + homogenised raw eggplant	93	10	66.1 (raw rice) 300 (vegetables) 2.5 (sesame oil) 1.5 (salt)	53.5	16 Healthy participants	Glucose and rice/4 h
477	^106^	China	Cooked rice + homogenised cooked eggplant	78	8	66.1 (raw rice) 300 (vegetables) 2.5 (sesame oil) 1.5 (salt)	53.5	16 Healthy participants	Glucose and rice/4 h
478	^107^	China	White rice cooked for 30 min	83	9	230 (66.1 g raw rice)	50	10 Healthy participants	Glucose and white rice/2 h
479	^107^	China	Waxy black rice cooked for 30 min	100	10	230 (66.1 g raw rice)	50	10 Healthy participants	Glucose and white rice/2 h
480	^107^	China	Waxy black rice cooked for 60 min	109	12	230 (66.1 g raw rice)	50	10 Healthy participants	Glucose and white rice/2 h
481	^107^	China	Foxtail millet cooked for 30 min	93	8	230 (72.3 g of millet)	50	10 Healthy participants	Glucose and White rice/2 h
482	^107^	China	Foxtail millet cooked for 60 min	89	6	230 (72.3 g of millet)	50	10 Healthy participants	Glucose and white rice/2 h
483	^107^	China	Adlay cooked for 30 min	91	10	230 (75.0 g of adlay)	50	10 Healthy participants	Glucose and white rice/2 h
484	^107^	China	Adlay cooked for 60 min	100	11	230 (75.0 g of adlay)	50	10 Healthy participants	Glucose and white rice/2 h
485	^107^	China	Dried lily bulb cooked for 30 min	83	9	230 (74.0 g of dried lily bulb)	50	10 Healthy participants	Glucose and white rice/2 h
486	^107^	China	Dried lily bulb cooked for 60 min	85	7	230 (74.0 g of dried lily bulb)	50	10 Healthy participants	Glucose and white rice/2 h
487	^107^	China	Lotus seed cooked for 30 min	45	5	230 (77.6 g of lotus seed)	50	10 Healthy participants	Glucose and White rice/2 h
488	^107^	China	Lotus seed cooked for 60 min	51	7	230 (77.6 g of lotus seed)	50	10 Healthy participants	Glucose and white rice/2 h
489	^107^	China	Adzuki bean cooked for 40 min	21	4	230 (83.1 g of adzuki bean)	50	10 Healthy participants	Glucose and white rice/2 h
490	^107^	China	Adzuki bean cooked for 70 min	29	4	230 (83.1 g of adzuki bean)	50	10 Healthy participants	Glucose and white rice/2 h
491	^108^	Taiwan, China	Brown rice	82	0.22	NA	50	10 Healthy participants	White bread/2 h
492	^108^	Taiwan, China	Taro	69	0.35	NA	50	10 Healthy participants	White bread/2 h
493	^108^	Taiwan, China	Adlay	55	0.4	NA	50	10 Healthy participants	White bread/2 h
494	^108^	Taiwan, China	Mung bean noodles	28	0.5	NA	50	10 Healthy participants	White bread/2 h
495	^108^	Taiwan, China	Yam	52	0.25	NA	50	10 Healthy participants	White bread/2 h
496	^109^	Taiwan	Brown rice (Taikeng 9)	49.8	4.3	NA	50	15 Healthy participants	Glucose/2 h
497	^109^	Taiwan	Brown rice (Taikeng 9)	70.8	4.3	NA	50	15 Healthy participants	White bread/2 h
498	^109^	Taiwan	Brown rice (Taichung Sen 10)	51	4.9	NA	50	15 Healthy participants	Glucose/2 h
499	^109^	Taiwan	Brown rice (Taichung Sen 10)	73	4.7	NA	50	15 Healthy participants	White bread/2 h
500	^109^	Taiwan	White rice (TRGC9152)	52.2	6.3	NA	50	15 Healthy participants	Glucose/2 h
501	^109^	Taiwan	White rice (TRGC9152)	73.1	5.7	NA	50	15 Healthy participants	White bread/2 h
502	^109^	Taiwan	White rice (IR50)	55.6	4	NA	50	15 Healthy participants	Glucose/2 h
503	^109^	Taiwan	White rice (IR50)	77.3	4.1	NA	50	15 Healthy participants	White bread/2 h
504	^109^	Taiwan	White rice (Taichung Sen 17)	47.3	4.7	NA	50	15 Healthy participants	Glucose/2 h
505	^109^	Taiwan	White rice (Taichung Sen 17)	71.7	4.2	NA	50	15 Healthy participants	White bread/2 h
506	^109^	Taiwan	White rice (Taikeng 9)	60.5	5.4	NA	50	15 Healthy participants	Glucose/2 h
507	^109^	Taiwan	White rice (Taikeng 9)	87.5	4.3	NA	50	15 Healthy participants	White bread/2 h
508	^109^	Taiwan	White rice (Taiching Sen 10)	55.6	3.2	NA	50	15 Healthy participants	Glucose/2 h
509	^109^	Taiwan	White rice (Taiching Sen 10)	82.5	5.5	NA	50	15 Healthy participants	White bread/2 h
510	^109^	Taiwan	White rice (Khazar)	62.4	6.9	NA	50	15 Healthy participants	Glucose/2 h
511	^109^	Taiwan	White rice (Khazar)	88.9	4.1	NA	50	15 Healthy participants	White bread/2 h
512	^110^	Taiwan	Steamed white rice	91.1	6.8	107	50	12 Healthy participants	Glucose/2 h
513	^110^	Taiwan	Rice porridge	98.4	8.1	290	50	12 Healthy participants	Glucose/2 h
514	^110^	Taiwan	Reheated overnight rice	90.6	6.6	107	50	12 Healthy participants	Glucose/2 h
515	^111^	Taiwan	Steamed white rice + 10 g of canola oil	90.3	2.1	117	50	12 healthy participants	White rice/2 h
516	^111^	Taiwan	Steamed white rice + 5 g dextrin fibre	89.3	2.6	112	50	12 Healthy participants	White rice/2 h
517	^111^	Taiwan	Steamed white rice + 10 g dextrin fibre	88.1	2.1	117	50	12 Healthy participants	White rice/2 h
518	^111^	Taiwan	Steamed white rice + 5 g gluten protein	84.9	1.7	112	50	12 Healthy participants	White rice/2 h
519	^111^	Taiwan	Steamed white rice + 10 g gluten protein	83.1	1.6	117	50	12 Healthy participants	White rice/2 h
520	^111^	Taiwan	Steamed white rice + 5 g gluten protein + 5 g dextrin fibre	88.5	1.9	117	50	12 Healthy participants	White rice/2 h
521	^111^	Taiwan	Steamed white rice + 5 g gluten protein + 10 g dextrin fibre	88.8	1.3	122	50	12 Healthy participants	White rice/2 h
522	^111^	Taiwan	Steamed white rice + 10 g gluten protein + 5 g dextrin fibre	86.2	1.3	122	50	12 Healthy participants	White rice/2 h
523	^111^	Taiwan	Steamed white rice + 10 g gluten protein+10 g dextrin fibre	86.1	1.7	127	50	12 Healthy participants	White rice/2 h
524	^111^	Taiwan	Steamed white rice + 5 g of canola oil + 5 g gluten protein	92	2.1	117	50	12 Healthy participants	White rice/2 h
525	^111^	Taiwan	Steamed white rice + 5 g of canola oil + 10 g gluten protein	91.9	2.3	122	50	12 Healthy participants	White rice/2 h
526	^111^	Taiwan	Steamed white rice + 10 g of canola oil + 5 g gluten protein	93.1	2.2	122	50	12 Healthy participants	White rice/2 h
527	^111^	Taiwan	Steamed white rice + 10 g of canola oil + 10 g gluten protein	91.3	1.7	127	50	12 Healthy participants	White rice/2 h
528	^111^	Taiwan	Steamed white rice + 5 g of canola oil + 5 g dextrin fibre	92.4	2.2	117	50	12 Healthy participants	White rice/2 h
529	^111^	Taiwan	Steamed white rice + 5 g of canola oil + 10 g dextrin fibre	94	2.2	122	50	12 Healthy participants	White rice/2 h
530	^111^	Taiwan	Steamed white rice + 10 g of canola oil + 5 g dextrin fibre	96	2	122	50	12 Healthy participants	White rice/2 h
531	^111^	Taiwan	Steamed white rice + 10 g of canola oil + 10 g dextrin fibre	96.3	2.2	127	50	12 Healthy participants	White rice/2 h
532	^111^	Taiwan	Steamed white rice + 5 g gluten protein + 5 g dextrin fibre + 5 g canola oil	92	2.6	122	50	12 Healthy participants	White rice/2 h
533	^111^	Taiwan	Steamed white rice + 5 g gluten protein + 10 g dextrin fibre +5 g canola oil	92.4	1.6	127	50	12 Healthy participants	White rice/2 h
534	^111^	Taiwan	Steamed white rice + 10 g gluten protein + 5 g dextrin fibre + 5 g canola oil	91.5	1.9	127	50	12 Healthy participants	White rice/2 h
535	^111^	Taiwan	Steamed white rice + 10 g gluten protein + 10 g dextrin fibre + 5 g canola oil	89	2.2	132	50	12 Healthy participants	White rice/2 h
536	^111^	Taiwan	Steamed white rice + 5 g gluten protein + 5 g dextrin fibre + 10 g canola oil	94.2	2.3	127	50	12 Healthy participants	White rice/2 h
537	^111^	Taiwan	Steamed white rice + 5 g gluten protein + 10 g dextrin fibre +10 g canola oil	93.1	1.6	132	50	12 Healthy participants	White rice/2 h
538	^111^	Taiwan	Steamed white rice + 10 g gluten protein + 5 g dextrin fibre +0 g canola oil	95.8	1.4	132	50	12 Healthy participants	White rice/2 h
539	^111^	Taiwan	Steamed white rice + 10 g gluten protein + 10 g dextrin fibre + 10 g canola oil	88.6	1.9	137	50	12 Healthy participants	White rice/2 h
540	^112^	Hong Kong, China	Baked barbecued pork puff	55	8	161	50	15 Healthy participants	Glucose/2 h
541	^112^	Hong Kong, China	Fried rice in Yangzhou style	80	6	217	50	15 Healthy participants	Glucose/2 h
542	^112^	Hong Kong, China	Fried fritter	69	9	139	50	15 Healthy participants	Glucose/2 h
543	^112^	Hong Kong, China	‘Mai-Lai’ cake	61	8	114	50	15 Healthy participants	Glucose/2 h
544	^112^	Hong Kong, China	Tuna fish bun	46	4	139	50	15 Healthy participants	Glucose/2 h
545	^112^	Hong Kong, China	Sticky rice wrapped in lotus leaf	83	5	167	50	15 Healthy participants	Glucose/2 h
546	^112^	Hong Kong, China	Steamed glutinous rice roll	89	8	109	50	15 Healthy participants	Glucose/2 h
547	^112^	Hong Kong, China	‘Pineapple’ bun	65	8	91	50	15 Healthy participants	Glucose/2 h
548	^112^	Hong Kong, China	Jam and peanut butter toast	72	8	106	50	15 Healthy participants	Glucose/2 h
549	^112^	Hong Kong, China	Fried rice noodles with sliced beef	66	7	250	50	15 Healthy participants	Glucose/2 h
550	^112^	Hong Kong, China	Egg tart	45	3	143	50	15 Healthy participants	Glucose/2 h
551	^112^	Hong Kong, China	Plain steamed vermicelli roll	90	8	238	50	15 Healthy participants	Glucose/2 h
552	^112^	Hong Kong, China	Green bean dessert	54	6	333	50	15 Healthy participants	Glucose/2 h
553	^112^	Hong Kong, China	Barbecue pork bun	69	9	119	50	15 Healthy participants	Glucose/2 h
554	^112^	Hong Kong, China	Red bean dessert	75	8	263	50	15 Healthy participants	Glucose/2 h
555	^112^	Hong Kong, China	Moon cakes	56	7	80	50	15 Healthy participants	Glucose/2 h
556	^112^	Hong Kong, China	Glutinous rice ball	61	10	115	50	15 Healthy participants	Glucose/2 h
557	^112^	Hong Kong, China	Chinese herbal jelly	47	3	333	50	15 Healthy participants	Glucose/2 h
558	^112^	Hong Kong, China	Instant sweet milky bun	67	5	114	50	15 Healthy participants	Glucose/2 h
559	^112^	Hong Kong, China	Frozen sweet milky bun	72	8	114	50	15 Healthy participants	Glucose/2 h
560	^112^	Hong Kong, China	Fried rice vermicelli in Singapore style	54	6	333	50	15 Healthy participants	Glucose/2 h
561	Chen et al., 2010)	Hong Kong, China	Fried rice vermicelli in Singapore style	69	8	167	50	15 Healthy participants	Glucose/2 h
562	^112^	Hong Kong, China	Salted meat rice dumpling	58	9	200	50	15 Healthy participants	Glucose/2 h
563	^112^	Hong Kong, China	Salted meat rice dumpling	81	7	100	50	15 Healthy participants	Glucose/2 h
564	^112^	Hong Kong, China	Spring roll	50	5	114	50	15 Healthy participants	Glucose/2 h
565	^113^	Hong Kong	Jianxi rice vermicelli	56	7	63.3	50	23 Healthy participants	Glucose/2 h
566	^113^	Hong Kong	Sau tao Bejing noodles	61	5	69.2	50	23 Healthy participants	Glucose/2 h
567	^113^	Hong Kong	Taiwan vermicelli	68	12	54.4	50	23 Healthy participants	Glucose/2 h
568	^113^	Hong Kong	Sau tao chicken-flavoured Sichuan spicy noodles	65	4	75.4	50	23 Healthy participants	Glucose/2 h
569	^113^	Hong Kong	Doll fried noodles	88	8	105.2	50	23 Healthy participants	Glucose/2 h
570	^113^	Hong Kong	Garden milk bar bun	73	8	105	50	23 Healthy participants	Glucose/2 h
571	^113^	Hong Kong	Linola seed bread	90	11	116.8	50	23 Healthy participants	Glucose/2 h
572	^114^	West India	Round leaf yellow yam, boiled	68	3	223.02	50	10 Healthy participants	Glucose/2 h
573	^114^	West India	Round leaf yellow yam, roasted	80	7	186.43	50	10 Healthy participants	Glucose/2 h
574	^114^	West India	Negro yam, boiled	73	4	235.07	50	10 Healthy participants	Glucose/2 h
575	^114^	West India	Negro yam, roasted	73	6	194.25	50	10 Healthy participants	Glucose/2 h
576	^114^	West India	Lucea yam, boiled	74	7	274.42	50	10 Healthy participants	Glucose/2 h
577	^114^	West India	Lucea yam, roasted	77	5	198.18	50	10 Healthy participants	Glucose/2 h
578	^114^	West India	White yam, boiled	75	6	239.00	50	10 Healthy participants	Glucose/2 h
579	^114^	West India	White yam, roasted	80	6	214.13	50	10 Healthy participants	Glucose/2 h
580	^114^	West India	Sweet yam, boiled	79	4	297.97	50	10 Healthy participants	Glucose/2 h
581	^114^	West India	Sweet yam, roasted	82	7	192.53	50	10 Healthy participants	Glucose/2 h
582	^114^	West India	Sweet potato, boiled	46	5	234.63	50	10 Healthy participants	Glucose/2 h
583	^114^	West India	Sweet potato, roasted	82	5	167.79	50	10 Healthy participants	Glucose/2 h
584	^114^	West India	Sweet potato, baked	94	8	167.79	50	10 Healthy participants	Glucose/2 h
585	^114^	West India	Sweet potato, fried	76	7	167.79	50	10 Healthy participants	Glucose/2 h
586	^114^	West India	Irish potato, boiled	59	4	230.95	50	10 Healthy participants	Glucose/2 h
587	^114^	West India	Irish potato, baked	83	6	249.63	50	10 Healthy participants	Glucose/2 h
588	^114^	West India	Irish potato, fried	70	6	249.63	50	10 Healthy participants	Glucose/2 h
589	^114^	West India	Dasheen, boiled	72	5	279.30	50	10 Healthy participants	Glucose/2 h
590	^114^	West India	Coco yam, boiled	61	5	482.63	50	10 Healthy participants	Glucose/2 h
591	^114^	West India	Pumpkin, boiled	66	4	223.81	50	10 Healthy participants	Glucose/2 h
592	^114^	West India	Breadfruit, boiled	47	5	276.55	50	10 Healthy participants	Glucose/2 h
593	^114^	West India	Breadfruit, roasted	72	8	221.34	50	10 Healthy participants	Glucose/2 h
594	^114^	West India	Green banana, boiled	37	5	225.23	50	10 Healthy participants	Glucose/2 h
595	^114^	West India	Green banana, fried	35	3	195.31	50	10 Healthy participants	Glucose/2 h
596	^114^	West India	Green plantain, boiled	39	4	259.20	50	10 Healthy participants	Glucose/2 h
597	^114^	West India	Green plantain, fried	40	3	175.93	50	10 Healthy participants	Glucose/2 h
598	^114^	West India	Ripe plantain, boiled	66	2	308.64	50	10 Healthy participants	Glucose/2 h
599	^114^	West India	Ripe plantain, fried	90	6	211.60	50	10 Healthy participants	Glucose/2 h
600	^115^	India	Biscuits (45% foxtail millet + 55% refined wheat flour)	50.8	27.9 (SD)	90	50	13 Healthy participants	Glucose/2.5 h
601	^115^	India	Biscuits (45% barnyard millet + 55% refined wheat flour)	68	60.3 (SD)	96	50	13 Healthy participants	Glucose/2.5 h
602	^115^	India	Biscuits (crude refined wheat flour)	68	52.8 (SD)	90	50	13 Healthy participants	Glucose/2.5 h
603	^116^	India	Papaya bar (control)	65	NA	64	50	15 Healthy participants	Glucose/2 h
604	^116^	India	Papaya bar (treated with inulin and fructooligosaccharides)	54	NA	65	50	15 Healthy participants	Glucose/2 h
605	^117^	India	Banana (yallakki)	43	NA	120	25	10 Healthy participants	Glucose/2 h
606	^117^	India	Mango (Raspuri)	35	NA	120	25	10 Healthy participants	Glucose/2 h
607	^117^	India	Papaya	19	NA	120	29	10 Healthy participants	Glucose/2 h
608	^117^	India	Orange	52	NA	120	10	10 Healthy participants	Glucose/2 h
609	^117^	India	Guava	78	NA	120	11.5	10 Healthy participants	Glucose/2 h
610	^117^	India	Chikku	73	NA	120	29	10 Healthy participants	Glucose/2 h
611	^117^	India	Jackfruit	63	NA	120	28.8	10 Healthy participants	Glucose/2 h
612	^117^	India	Watermelon	37	NA	120	6	10 Healthy participants	Glucose/2 h
613	^117^	India	Pineapple	19	NA	120	10	10 Healthy participants	Glucose/2 h
614	^117^	India	Apple	45	NA	120	16	10 Healthy participants	Glucose/2 h
615	^118^	India	Roasted Amaranth Grains flour chapatti	84.83	50	117.5	50	50 NIDDM participants	Glucose/2 h
616	^118^	India	Boiled Amaranth Grains flour chapatti	111.83	75	118	50	50 NIDDM participants	Glucose/2 h
617	^118^	India	Popped Amaranth Grains flour chapatti	44	25.08	116.34	50	50 NIDDM participants	Glucose/2 h
618	^118^	India	Raw Amaranth Grains flour chapatti	102.3	76.4	121.39	50	50 NIDDM participants	Glucose/2 h
619	^119^	India	Sona Masuri (parboiled rice)	72	4.5	235	50	30 Healthy participants	Glucose/2 h
620	^119^	India	Ponni (parboiled rice)	70.2	3.6	236	50	30 Healthy participants	Glucose/2 h
621	^119^	India	Surti Kolam (parboiled rice)	77	4.0	259	50	30 Healthy participants	Glucose/2 h
622	^120^	India	Burfi (made with 43% foxtail millet, 57% bengal gram flour)	37.5	18.5 (SD)	NA	50	10 Healthy participants	Glucose/2.5 h
623	^120^	India	Burfi (made with 43% barnyard millet, 57% bengal gram flour)	45.0	14.5 (SD)	NA	50	10 Healthy participants	Glucose/2.5 h
624	^120^	India	Burfi (made with 100% bengal gram flour)	43.0	14.9	NA	50	10 Healthy participants	Glucose/2.5 h
625	^121^	India	Namkeen sev (without dried bottle gourd pulp powder) [bengal gram flour + kidney bean flour (50:50)]	32.82	NA	NA	50	10 Healthy participants	Glucose/2 h
626	^121^	India	Namkeen sev (with dried bottle gourd pulp powder) [bengal gram flour + kidney bean flour + DBPP (40:40:20)]	21.83	NA	NA	50	10 Healthy participants	Glucose/2 h
627	^122^	India	Idli	67.11	3.25	70	50	10 Healthy participants	Glucose/2 h
628	^122^	India	Sewai upma	69.1	1.74	147	50	10 Healthy participants	Glucose/2 h
629	^122^	India	Idli (60% Kodo millet)	58.53	1.48	76	50	10 Healthy participants	Glucose/2 h
630	^122^	India	Sewai upma (60% Kodo millet)	65.49	1.01	150	50	10 Healthy participants	Glucose/2 h
631	^123^	India	Chapatti (whole wheat)	83.92	9.63 (SD)	NA	50	20 NIDDM participants	Glucose/2 h
632	^123^	India	Chapatti (whole wheat + rice bran-based)	68.34	11.49 (SD)	NA	50	20 NIDDM participants	Glucose/2 h
633	^124^	India	Indian branded basmati rice	54.93	1.07	NA	50	70 Healthy participants	Glucose/2 hr
634	^125^	India	Misi parantha	40.41	NA	NA	50	10 healthy participants	Glucose/2.5 h
635	^125^	India	Misi parantha (15% green gram husk)	32.54	NA	NA	50	10 Healthy participants	Glucose/2.5 h
636	^126^	India	Dal samosa (added 10% *Ficus religiosa* leaves)	35	NA	NA	50	25 Healthy participants	Glucose/2 h
637	^126^	India	Bati (added 5% *Ficus religiosa* bark)	53	NA	NA	50	25 Healthy participants	Glucose/2 h
638	^127^	India	Noodles (30% finger millet flour + refined wheat flour)	45.1	NA	64.97	50	10 Healthy participants	Glucose/2.5 h
639	^127^	India	Noodles (refined wheat flour)	62.6	NA	65.66	50	10 Healthy participants	Glucose/2.5 h
640	^128^	India	Refined wheat noodles	66.43	NA	NA	50	10 Healthy participants	Glucose/2 h
641	^128^	India	Refined wheat noodles (added bengal gram seed coat + broken rice)	56.13	NA	NA	50	10 Healthy participants	Glucose/2 h
642	^128^	India	Refined wheat noodles (added bengal gram broken + broken rice)	45.78	NA	NA	50	10 Healthy participants	Glucose/2 h
643	^129^	India	Biscuit (refined wheat flour)	68.70	NA	NA	50	10 Healthy participants	Glucose/2.5 h
644	^129^	India	Biscuit (refined wheat flour with 12% green gram husk)	46.26	NA	NA	50	10 Healthy participants	Glucose/2.5 h
645	^130^	India	Banana (Nendran)	87.29	NA	NA	50	20 Healthy participants	Glucose/2 h
646	^130^	India	Banana (Robusta)	81.55	NA	NA	50	20 Healthy participants	Glucose/2 h
647	^130^	India	Banana (Poovan)	83.36	NA	NA	50	20 Healthy participants	Glucose/2 h
648	^130^	India	Banana (Chenkadali)	82.23	NA	NA	50	20 Healthy participants	Glucose/2 h
649	^130^	India	Banana (Njalipoovan)	95.98	NA	NA	50	20 Healthy participants	Glucose/2 h
650	^131^	India	Little millet flakes (ready to cook)	52.11	NA	84	50	10 Healthy participants	Glucose/2.5 h
651	^132^	India	Sorghum multigrain roti	68	8.63	119	50	10 Healthy participants	Glucose/2 h
652	^132^	India	Sorghum coarse semolina upma	53	2.84	232	50	10 Healthy participants	Glucose/2 h
653	^132^	India	Sorghum fine semolina upma	56	9.83	252	50	10 Healthy participants	Glucose/2 h
654	^132^	India	Sorghum flakes poha	45	5.27	277	50	10 Healthy participants	Glucose/2 h
655	^132^	India	Sorghum pasta	46	6.47	330	50	10 Healthy participants	Glucose/2 h
656	^132^	India	Sorghum biscuits	54	6.3	75	50	10 Healthy participants	Glucose/2 h
657	^132^	India	Wheat roti	64	9.24	119	50	10 Healthy participants	Glucose/2 h
658	^132^	India	Wheat coarse semolina upma	58	6.85	232	50	10 Healthy participants	Glucose/2 h
659	^132^	India	Wheat fine semolina upma	67	10.8	252	50	10 Healthy participants	Glucose/2 h
660	^132^	India	Rice flakes poha	74	4.87	277	50	10 Healthy participants	Glucose/2 h
661	^132^	India	Wheat pasta	72	6.51	330	50	10 Healthy participants	Glucose/2 h
662	^132^	India	Wheat biscuits	57	11.4	75	50	10 Healthy participants	Glucose/2 h
663	^133^	India	Khichdi (barnyard millet)	34.96	1.22 (SD)	NA	50	10 Healthy participants	Glucose/2.5 h
664	^133^	India	Rice khichdi	62.5	1.38 (SD)	NA	50	10 Healthy participants	Glucose/2.5 h
665	^134^	India	High fibre white rice	61.3	2.8	67 g (raw) 1:2 water	50	39 Healthy participants	Glucose/2 h
666	^134^	India	White rice	79.2	4.8	65 g (raw) 1:2 water	50	40 Healthy participants	Glucose/2 h
667	^135^	India	Dosa (rice-based)	77.86	NA	140	50	10 Healthy participants	White bread/2 h
668	^135^	India	Dosa (foxtail millet-based)	59.25	NA	290	50	10 Healthy participants	White bread/2 h
669	^136^	India	Brown ragi roti	61.0	5.77	69.44	50	10 Healthy participants	Glucose/2 h
670	^136^	India	White ragi roti	67.3	2.78	69.44	50	10 Healthy participants	Glucose/2 h
671	^136^	India	Brown ragi roti + curry leaf powder (CLP)	56.2	5.56	64.58 (flour) 5 (CLP)	50	10 Healthy participants	Glucose/2 h
672	^136^	India	White ragi flour roti + curry leaf powder (CLP)	62.5	4.23	64.58 (flour) 5 (CLP)	50	10 Healthy participants	Glucose/2 h
673	^137^	India	Wheat chapatti	48.37	20.59 (SD)	72.05 wheat flour	50	10 Healthy participants	Glucose/2 h
674	^137^	India	Wheat chapatti enriched with carrot powder	53.48	16.91 (SD)	72.22 wheat flour 15% carrot powder	50	10 Healthy participants	Glucose/2 h
675	^137^	India	Dalia	38.05	27.04 (SD)	10 g dalia 10 g moong dal 280 ml water	50	10 Healthy participants	Glucose/2 h
676	^137^	India	Salty enriched dalia (with carrot grits)	49.81	25.69 (SD)	13.34 g daliav 6.66 g moong dal 10.73 g carrot grits 279 ml water	50	10 Healthy participants	Glucose/2 h
677	^138^	India	Chakli (added 5% kale powder)	48.86	NA	NA	50	30 Healthy participants	Glucose/2 h
678	^138^	India	Twisters (added 10% kale powder)	46.44	NA	NA	50	30 Healthy participants	Glucose/2 h
679	^139^	India	Biscuits [(refined wheat flour, barley flour and soy flour (25:50:25)]	38.68	NA	108.5	50	10 Healthy participants	Glucose/2 h
680	^139^	India	Biscuits (100% refined wheat flour)	83.99	NA	94	50	10 Healthy participants	White bread/2 h
681	^140^	India	Extruded snack (whole-wheat flour, barley and chickpea, 50:25:25)	48.77	NA	67.5	50	10 Healthy participants	Glucose/2 h
682	^140^	India	Extruded snack (100% whole-wheat flour)	69.68	NA	64	50	10 Healthy participants	Glucose/2 h
683	^141^	India	Brown rice	57.6	6.8	NA	50	12 Healthy participants	Glucose/2 h
684	^141^	India	Minimally polished/under milled rice with 2.3% degree of polish (≈hand pounded rice)	73	5.4	NA	50	12 Healthy participants	Glucose/2 h
685	^141^	India	Fully polished white rice (WR) with 9.7% degree of polish	79.6	6.8	NA	50	12 Healthy participants	Glucose/2 h
686	^142^	India	Uzhunnu vada	21.54	NA	172	50	11 Healthy participants	Glucose/2 h
687	^142^	India	Tapioca	83.57	NA	135	50	11 healthy participants	Glucose/2 h
688	^142^	India	Dosa	55.80	NA	120	50	11 Healthy participants	Glucose/2 h
689	^142^	India	Puttu	62.68	NA	141	50	11 Healthy participants	Glucose/2 h
690	^142^	India	Plaintain (unripe)	73.9	NA	422	50	11 Healthy participants	Glucose/2 h
691	^142^	India	Chapathi	54.43	NA	110	50	11 Healthy participants	Glucose/2 h
692	^142^	India	Poori	58.53	NA	121	50	11 Healthy participants	Glucose/2 h
693	^142^	India	Idiyappam	59.41	NA	131	50	11 Healthy participants	Glucose/2 h
694	^142^	India	Appam	59.94	NA	122	50	11 Healthy participants	Glucose/2 h
695	^142^	India	Yam	55.53	NA	282	50	11 Healthy participants	Glucose/2 h
696	^142^	India	Porotta	37.98	NA	121	50	11 Healthy participants	Glucose/2 h
697	^142^	India	Semolina upma	62.37	NA	142	50	11 Healthy participants	Glucose/2 h
698	^142^	India	Idli	62.45	NA	149	50	11 Healthy participants	Glucose/2 h
699	^143^	India	Thepla	57.77	NA	NA	50	30 Healthy participants	Glucose/2 h
700	^143^	India	Thepla (2% ashwagandha root powder)	37.30	NA	NA	50	30 Healthy participants	Glucose/2 h
701	^144^	India	Maize	75.15	0.60 (SD)	NA	50	10 Healthy participants	Glucose/2 h
702	^144^	India	Boiled maize (with whole bengal gram)	68.72	0.86 (SD)	NA	50	10 Healthy participants	Glucose/2 h
703	^144^	India	Alkali-treated maize (with whole bengal gram)	69.01	0.66 (SD)	NA	50	10 Healthy participants	Glucose/2 h
704	^144^	India	Roasted maize (with whole bengal gram)	72.15	0.60 (SD)	NA	50	10 Healthy participants	Glucose/2 h
705	^145^	India	DiaBliss herbal sugar (DHS)	46.5	NA	50	50	16 Healthy participants	Glucose/2 h
706	^146^	India	Mixed mini meal: wheat, pearl barley and Bengal gram flour (besan) mix with chana whole (unhusked chana + curd)	71.9	7.4	NA	50	12 Healthy participants	Glucose/2 h
707	^147^	India	Upma (added decorticated finger millet with lower degree of polish)	84.7	8.2	NA	50	16 Healthy participants	Glucose/2 h
708	^147^	India	Upma (added finger millet flakes)	82.3	6.8	NA	50	16 Healthy participants	Glucose/2 h
709	^147^	India	Upma (added finger millet vermicelli)	65.5	5.5	NA	50	16 Healthy participants	Glucose/2 h
710	^147^	India	Finger millet extruded snack	65	6.6	NA	50	12 Healthy participants	Glucose/2 h
711	^148^	India	Roti (whole-wheat flour)	44.6	NA	55	50	30 Healthy participants	Dextrose/2 h
712	^148^	India	Chappati (multigrain flour)	28.4	NA	84	50	30 Healthy participants	Dextrose/2 h
713	^149^	India	Green jackfruit (freeze-dried) porridge	65	5	NA	25 or 50	10 Healthy participants	Glucodin/2 h
714	^150^	India	Kashi 7 whole-grain ‘pilaf’	58.9	5.1	160	50	14 Healthy participants	Glucose/2 h
715	^150^	India	Uncle Ben’s whole-grain fast and natural instant brown rice	87.8	6.8	193	50	14 Healthy participants	Glucose/2 h
716	^150^	India	Refined maize ugali flour	71.4	5.1	161	50	14 Healthy participants	Glucose/2 h
717	^150^	India	Whole maize ugali flour	74.7	6.5	164	50	14 Healthy participants	Glucose/2 h
718	^151^	India	Millet-based roti	53	NA	NA	50	10 Healthy participants	Glucose/2 h
719	^151^	India	Millet-based dosa	37	NA	NA	50	10 Healthy participants	Glucose/2 h
720	^151^	India	Millet-based dumpling	48	NA	NA	50	10 Healthy participants	Glucose/2 h
721	^152^	India	Preserved coconut sugar	52.47	NA	62.5	50	15 Healthy participants	Glucose/2 h
722	^153^	Sri Lanka	White sliced bread	77	6	114	50	10 Healthy participants	Glucose/2 h
723	^153^	Sri Lanka	Wholemeal bread	77	6	128	50	10 Healthy participants	Glucose/2 h
724	^153^	Sri Lanka	Ordinary white bread	80	4	121	50	10 Healthy participants	Glucose/2 h
725	^153^	Sri Lanka	Wholemeal bread and lentil curry	61	6	Bread: 83 Curry: 150	50	10 Healthy participants	Glucose/2 h
726	^153^	Sri Lanka	White sliced bread	100	NA	114	50	10 Healthy participants	White bread/2 h
727	^153^	Sri Lanka	Wholemeal bread	103	10	128	50	10 Healthy participants	White bread/2 h
728	^153^	Sri Lanka	Ordinary white bread	114	11	121	50	10 Healthy participants	White bread/2 h
729	^153^	Sri Lanka	Wholemeal bread and lentil curry	87	6	Bread: 83 Curry: 150	50	10 Healthy participants	White bread/2 h
730	^154^	Sri Lanka	Wheat flour roti	72	6	NA	50	10 Healthy participants	White bread/2 h
731	^154^	Sri Lanka	Rice flour roti	69	7	NA	50	10 Healthy participants	White bread/2 h
732	^154^	Sri Lanka	Kurakkan flour roti	70	8	NA	50	10 Healthy participants	White bread/2 h
733	^154^	Sri Lanka	Atta flour roti	67	9	NA	25	10 Healthy participants	White bread/2 h
734	^154^	Sri Lanka	Wheat flour pittu	101	8	NA	25	10 Healthy participants	White bread/2 h
735	^154^	Sri Lanka	Rice flour pittu	103	7	NA	25	10 Healthy participants	White bread/2 h
736	^154^	Sri Lanka	Kurakkan flour pittu	85	6	NA	25	10 Healthy participants	White bread/2 h
737	^154^	Sri Lanka	Boiled chickpea	29	5	NA	25	10 Healthy participants	White bread/2 h
738	^154^	Sri Lanka	Boiled mung bean	57	6	NA	25	10 Healthy participants	White bread/2 h
739	^154^	Sri Lanka	Boiled cowpea	49	8	NA	25	10 Healthy participants	White bread/2 h
740	^154^	Sri Lanka	Olu-milk rice	91	8	NA	25	10 Healthy participants	White bread/2 h
741	^154^	Sri Lanka	Breadfruit	65	7	NA	25	10 Healthy participants	White bread/2 h
742	^154^	Sri Lanka	Hopperss	120	8	NA	25	10 Healthy participants	White bread/2 h
743	^155^	Sri Lanka	Parboiled rice with green curry	47.47	11.20	375	75	20 Healthy participants	Glucose/2 h
744	^155^	Sri Lanka	Parboiled rice with gravy	56.30	9.31	355	75	20 Healthy participants	Glucose/2 h
745	^155^	Sri Lanka	Parboiled rice with green curry and gravy	54.67	10.03	405	75	20 Healthy participants	Glucose/2 h
746	^155^	Sri Lanka	‘Kurakkan pittu’ with green curry	57.51	5.52	262	75	20 Healthy participants	Glucose/2 h
747	^155^	Sri Lanka	‘Kurakkan pittu’ with gravy	63.25	8.86	242	75	20 Healthy participants	Glucose/2 h
748	^155^	Sri Lanka	‘Kurakkan pittu’ with green curry and gravy	59.25	5.49	292	75	20 Healthy participants	Glucose/2 h
749	^155^	Sri Lanka	‘Atta pittu’ with green curry	44.40	14.27	327	75	20 Healthy participants	Glucose/2 h
750	^155^	Sri Lanka	‘Atta pittu’ with gravy	50.80	9.35	307	75	20 Healthy participants	Glucose/2 h
751	^155^	Sri Lanka	‘Atta pittu’ with green curry and gravy	46.29	8.90	357	75	20 Healthy participants	Glucose/2 h
752	^156^	Sri Lanka	Chickpea meal	40	7	186	25	11 T2DM participants	White bread/3 h
753	^156^	Sri Lanka	Red rice meal + accompaniments	64	11	149	25	11 T2DM participants	White bread/3 h
754	^156^	Sri Lanka	Atta roti meal + accompaniments	88	9	85	25	11 T2DM participants	White bread/3 h
755	^157^	Sri Lanka	Rice with lentil curry, boiled egg, coconut gravy and *Trichosanthes cucumerin*a (snake gourd) salad	61	5	285 + 30 ml coconut gravy	50	10 Healthy participants	Bread/2 h
756	^156^	Sri Lanka	Chickpea meal	40	7	186	25	11 T2DM participants	White bread/3 h
757	^156^	Sri Lanka	Red rice meal + accompaniments	64	11	85	25	11 T2DM participants	White bread/3 h
758	^156^	Sri Lanka	Atta roti meal + accompaniments	88	9	149	25	11 T2DM participants	White bread/3 h
759	^158^	Sri Lanka	Banana (Silk)	61	5	190	50	10 Healthy participants	Glucose/2 h
760	^158^	Sri Lanka	Banana (Mysore)	61	6	220	50	10 Healthy participants	Glucose/2 h
761	^158^	Sri Lanka	Banana (Gros Michel)	67	7	270	50	10 Healthy participants	Glucose/2 h
762	^158^	Sri Lanka	Banana (Pisang Awak)	69	9	220	50	10 Healthy participants	Glucose/2 h
763	^159^	Sri Lanka	White rice	66.61	9.86 (SD)	286.04	75	22 Healthy participants	Glucose/2.5 h
764	^159^	Sri Lanka	Brown rice	60.24	8.16 (SD)	338	75	22 Healthy participants	Glucose/2.5 h
765	^159^	Sri Lanka	Parboiled rice	55.97	6.01 (SD)	324.67	75	22 Healthy participants	Glucose/2.5 h
766	^159^	Sri Lanka	Pittu (from cereal flour)	43.74	9.09 (SD)	166.55	75	22 Healthy participants	Glucose/2.5 h
767	^159^	Sri Lanka	‘String hopper’ (from cereal flour)	50.01	7.06 (SD)	200	75	22 Healthy participants	Glucose/2.5 h
768	^159^	Sri Lanka	Cassava (tuber)	78.67	7.30 (SD)	232.56	75	22 Healthy participants	Glucose/2.5 h
769	^159^	Sri Lanka	Green gram (legume)	31.43	6.96 (SD)	294.92	75	22 Healthy participants	Glucose/2.5 h
770	^159^	Sri Lanka	Chickpea (legume)	33.27	6.23 (SD)	253.2	75	22 Healthy participants	Glucose/2.5 h
771	^160^	Sri Lanka	Kathali	54.45	9.26 (SD)	325.95	75	20 Healthy participants	Glucose/2 h
772	^160^	Sri Lanka	Kappal	50.43	5.79 (SD)	314.33	75	20 Healthy participants	Glucose/2 h
773	^160^	Sri Lanka	Itharai	48.47	10.13 (SD)	277.16	75	20 Healthy participants	Glucose/2 h
774	^160^	Sri Lanka	Jackfruit	65.36	8.00 (SD)	578.70	75	20 Healthy participants	Glucose/2 h
775	^160^	Sri Lanka	Papaya	34.90	12.78 (SD)	903.60	75	20 Healthy participants	Glucose/2 h
776	^161^	Sri Lanka	Coconut milk porridge (*Cocos nucifera*)	31	5	Coconut milk porridge was made with rice and coconut milk in 25:90 ratio	25	10 Healthy participants	Glucose/2 h
777	^161^	Sri Lanka	Rice porridge	46	17	Rice porridge was prepared with rice and water (25:90)	25	10 Healthy participants	Glucose/2 h
778	^161^	Sri Lanka	*Murraya koenigii* Spreng (Karapincha)	44	8	All porridges were cooked until final volume of 300 ml (in the porridge, leaves: coconut milk:rice = 13:90:25)	25	10 Healthy participants	Glucose/2 h
779	^161^	Sri Lanka	*Hemidesmus indicus* (Iramusu)	40	8	NA	25	10 Healthy participants	Glucose/2 h
780	^161^	Sri Lanka	*Aegle marmelos* (Beli)	50	8	NA	25	10 Healthy participants	Glucose/2 h
781	^161^	Sri Lanka	*Coreopsis auriculata* Linn. (Ranawara)	77	12	NA	25	10 Healthy participants	Glucose/2 h
782	^161^	Sri Lanka	*Clitoria ternatea* Linn. (Ela katarolu)	53	10	NA	25	10 Healthy participants	Glucose/2 h
783	^161^	Sri Lanka	*Cardiospermum halicacabum* (Wel Penela)	46	8	NA	25	10 Healthy participants	Glucose/2 h
784	^161^	Sri Lanka	*Alphonsea zeylanica* Linn. (Yaki narang)	52	13	NA	25	10 Healthy participants	Glucose/2 h
785	^161^	Sri Lanka	*Cannabis indica* (Kowakka)	49	8	NA	25	10 Healthy participants	Glucose/2 h
786	^161^	Sri Lanka	*Osbeckia octandra* (Heen bovitiya)	55	7	NA	25	10 Healthy participants	Glucose/2 h
787	^161^	Sri Lanka	*Aerva lanata* (Polpala)	32	5	NA	25	10 Healthy participants	Glucose/2 h
788	^161^	Sri Lanka	*Asparagus racemosus* (haathawaariya)	37	4	NA	25	10 Healthy participants	Glucose/2 h
789	^161^	Sri Lanka	*Scoparia dulcis* (Wal koththamalli)	39	8	NA	25	10 Healthy participants	Glucose/2 h
790	^161^	Sri Lanka	Rice with lentil curry, boiled egg, coconut gravy and *Centella asiatica* (gotukola) leaves salad	63	6	285 + 30 ml coconut gravy	50	10 Healthy participants	Bread/2 h
791	^161^	Sri Lanka	Rice with lentil curry, boiled egg, coconut gravy and *Lasia spinosa* (kohila) salad	57	5	285 + 30 ml coconut gravy	50	10 Healthy participants	Bread/2 h
792	^162^	Sri Lanka	Brown rice flour string hoppers + beans curry	39.93	8.14	NA	50	30 Healthy participants	Glucose/2 h
793	^162^	Sri Lanka	White rice flour string hoppers + beans curry	41.96	9.86	NA	50	30 Healthy participants	Glucose/2 h
794	^162^	Sri Lanka	Brown rice flour string hoppers + lentil curry	44.30	9.25	NA	50	30 Healthy participants	Glucose/2 h
795	^162^	Sri Lanka	White rice flour string hoppers + lentil curry	53.46	9.57	NA	50	30 Healthy participants	Glucose/2 h
796	^162^	Sri Lanka	Brown rice flour string hoppers + fish curry	45.26	9.25	NA	50	30 Healthy participants	Glucose/2 h
797	^162^	Sri Lanka	White rice flour string hoppers + fish curry	56.13	9.94	NA	50	30 Healthy participants	Glucose/2 h
798	^162^	Sri Lanka	Brown rice flour string hoppers + coconut gravy + polsambol	50.46	9.74	NA	50	30 Healthy participants	Glucose/2 h
799	^162^	Sri Lanka	White rice flour string hoppers + coconut gravy + polsambol	69.20	9.47	NA	50	30 Healthy participants	Glucose/2 h
800	^163^	Sri Lanka	Pittu (made with 25% soy flour and 75% rice flour)	35.5	9.8	330 (4 medium size)	50	13 Healthy participants	Glucose/2 h
801	^163^	Sri Lanka	Pittu with vegetable curry	30.2	6.5 (SD)	386.2	50	13 Healthy participants	Glucose/2 h
802	^163^	Sri Lanka	Rotti (made with 25% soy flour and 75% rice flour)	36.04	8.1 (SD)	244.1 (4 medium size)	50	13 Healthy participants	Glucose/2 h
803	^163^	Sri Lanka	Rotti with vegetable curry	31.15	4.6 (SD)	311.7	50	13 Healthy participants	Glucose/2 h
804	^163^	Sri Lanka	Wandu (made with 25% soy flour and 75% rice flour)	42.97	8.9 (SD)	400 (8 pcs)	50	13 Healthy participants	Glucose/2 h
805	^163^	Sri Lanka	Wandu with vegetable curry	36.46	5.4 (SD)	446.9	50	13 Healthy participants	Glucose/2 h
806	^163^	Sri Lanka	Hopper (made with 25% soy flour and 75% rice flour)	45.18	8.6 (SD)	340 (8 pcs)	50	13 Healthy participants	Glucose/2 h
807	^163^	Sri Lanka	Hopper with vegetable curry	38.3	5.6 (SD)	394.9	50	13 Healthy participants	Glucose/2 h
808	^163^	Sri Lanka	Thosai (made with 25% soy flour and 75% rice flour)	47.34	5.3 (SD)	353 (7 pcs)	50	13 Healthy participants	Glucose/2 h
809	^164^	Sri Lanka	Thosai and sambol	63.93	7.62	NA	75	20 Healthy participants	Glucose/2 h
810	^164^	Sri Lanka	Thosai, sambol and plantain	60.17	3.58	NA	75	20 Healthy participants	Glucose/2 h
811	^164^	Sri Lanka	Thosai and Sampar	71.90	4.73	NA	75	20 Healthy participants	Glucose/2 h
812	^164^	Sri Lanka	Thosai, sampar and plantain	68.57	4.18	NA	75	20 healthy participants	Glucose/2 h
813	^164^	Sri Lanka	Thosai, sambol and sampar	65.63	3.46	NA	75	20 Healthy participants	Glucose/2 h
814	^164^	Sri Lanka	Thosai, sambol, sampar and plantain	63.04	5.05	NA	75	20 Healthy participants	Glucose/2 h
815	^165^	Sri Lanka	Pakistani Basmati rice (rice cooker) + 20 g coconut sambol	64	12	147 g (cooked)	50	10 Healthy participants	Glucose/2 h
816	^165^	Sri Lanka	Indian Basmati rice (rice cooker) + 20 g coconut sambol	54	8 (SD)	151 g (cooked)	50	10 Healthy participants	Glucose/2 h
817	^165^	Sri Lanka	Pakistani Basmati rice (microwave) + 120 g coconut sambol	56	14 (SD)	147 g (cooked)	50	10 Healthy participants	Glucose/2 h
818	^165^	Sri Lanka	Indian Basmati rice (microwave) + 0 g coconut sambol	43	6 (SD)	151 g (cooked)	50	10 Healthy participants	Glucose/2 h
819	^166^	Sri Lanka	Parboiled rice	55.97	6.01	NA	75	20 Healthy participants	Glucose/2 h
820	^166^	Sri Lanka	White rice (Sampa)	66.61	9.86	NA	75	20 Healthy participants	Glucose/2 h
821	^166^	Sri Lanka	Brown rice	60.24	8.16	NA	75	20 Healthy participants	Glucose/2 h
822	^166^	Sri Lanka	String hoppers	50.01	7.06	NA	75	20 Healthy participants	Glucose/2 h
823	^166^	Sri Lanka	Pittu	43.74	9.09	NA	75	20 Healthy participants	Glucose/2 h
824	^166^	Sri Lanka	Parboiled rice + green leafy curry	47.5	NA	NA	75	20 Healthy participants	Glucose/2 h
825	^166^	Sri Lanka	Parboiled rice + gravy	56.3	NA	NA	75	20 Healthy participants	Glucose/2 h
826	^166^	Sri Lanka	Parboiled rice + green leafy curry and gravy	54.7	NA	NA	75	20 Healthy participants	Glucose/2 h
827	^166^	Sri Lanka	Pittu (kurakan flour) + green leafy curry	57.5	NA	NA	75	20 Healthy participants	Glucose/2 h
828	^166^	Sri Lanka	Pittu (kurakan flour) + gravy	63.3	NA	NA	75	20 Healthy participants	Glucose/2 h
829	^166^	Sri Lanka	Pittu (kurakan flour) + green leafy curry + gravy	59.3	NA	NA	75	20 Healthy participants	Glucose/2 h
830	^166^	Sri Lanka	Pittu (atta flour) + green leafy curry	44.4	NA	NA	75	20 Healthy participants	Glucose/2 h
831	^166^	Sri Lanka	Pittu (atta flour) + gravy	50.8	NA	NA	75	20 Healthy participants	Glucose/2 h
832	^166^	Sri Lanka	Pittu (atta flour) +green leafy curry + gravy	46.3	NA	NA	75	20 Healthy participants	Glucose/2 h
833	^166^	Sri Lanka	Boiled potato	65.2	6.56	NA	75	20 Healthy participants	Glucose/2 h
834	^166^	Sri Lanka	Boiled cassava	78.7	7.3	NA	75	20 Healthy participants	Glucose/2 h
835	^166^	Sri Lanka	Boiled chickpea	33.3	6.23	NA	75	20 Healthy participants	Glucose/2 h
836	^166^	Sri Lanka	Boiled green gram	31.4	6.96	NA	75	20 Healthy participants	Glucose/2 h
837	^166^	Sri Lanka	Wheat flour bread	68.59	NA	NA	75	20 Healthy participants	Glucose/2 h
838	^166^	Sri Lanka	Normal bun	67.3	NA	NA	75	20 Healthy participants	Glucose/2 h
839	^166^	Sri Lanka	Hard bun	52.78	NA	NA	75	20 Healthy participants	Glucose/2 h
840	^166^	Sri Lanka	Butter cake	64.72	NA	NA	75	20 Healthy participants	Glucose/2 h
841	^166^	Sri Lanka	Rusk	50.30	NA	NA	75	20 Healthy participants	Glucose/2 h
842	^166^	Sri Lanka	Idli and sambol	56.85	6.26	NA	75	20 Healthy participants	Glucose/2 h
843	^166^	Sri Lanka	Idli, sambol and plantain	51.10	6.57	NA	75	20 Healthy participants	Glucose/2 h
844	^166^	Sri Lanka	Idli and sampar	70.32	8.22	NA	75	20 Healthy participants	Glucose/2 h
845	^166^	Sri Lanka	Idli, sampar and plantain	67.45	7.87	NA	75	20 Healthy participants	Glucose/2 h
846	^166^	Sri Lanka	Idli, sambol and sampar	63.09	3.29	NA	75	20 Healthy participants	Glucose/2 h
847	^166^	Sri Lanka	Idli, sambol, sampar and plantain	61.30	3.09	NA	75	20 Healthy participants	Glucose/2 h
848	^167^	Sri Lanka	Roti (*Caryote urens*)	57	4	114	50	10 Healthy participants	White bread/2 h
849	^167^	Sri Lanka	Porridge (*Caryote urens*)	128	11	530	50	10 Healthy participants	White bread/2 h
850	^167^	Sri Lanka	Muffin (*Caryote urens*)	92	9	126	50	10 Healthy participants	White bread/2 h
851	^167^	Sri Lanka	Roti (*Cycas circanlis*)	66	6	135	50	10 Healthy participants	White bread/2 h
852	^167^	Sri Lanka	Pittu (*Cycas circanlis*)	72	4	145	50	10 Healthy participants	White bread/2 h
853	^167^	Sri Lanka	Pittu (*Vateria copalifera*)	67	7	215	50	10 Healthy participants	White bread/2 h
854	^168^	Sri Lanka	Skimmed milk powder with powdered oats and whey	12	2	NA	50	11 Healthy participants	Glucose/2 h
855	^169^	Sri Lanka	Red pigmented rice (Kaluheenati)	56.3	2.5	NA	50	12 Healthy participants	Glucose/2 h
856	^169^	Sri Lanka	Red pigmented rice (Wedaheenati)	52.5	1.5	NA	50	12 Healthy participants	Glucose/2 h
857	^169^	Sri Lanka	Red pigmented rice (Rathkaral)	62.0	3.1	NA	50	12 Healthy participants	Glucose/2 h
858	^169^	Sri Lanka	Red pigmented rice (Madathawalu)	64.0	2.5	NA	50	12 Healthy participants	Glucose/2 h
859	^170^	United Arab Emirates (UAE)	Rutab (dates)	47.2	17.4	101.8	50	11 Healthy participants	Glucose/2 h
860	^170^	United Arab Emirates (UAE)	Commercial tamer (dates)	35.5	9.7	66.7	50	11 Healthy participants	Glucose/2 h
861	^170^	United Arab Emirates (UAE)	Traditional dates	45.3	25.6	66.8	50	11 Healthy participants	Glucose/2 h
862	^171^	United Arab Emirates (UAE)	Fara’d (dates)	54.0	6.1	72.5	50	13 Healthy participants	Glucose/2 h
863	^171^	United Arab Emirates (UAE)	Fara’d (dates)	46.1	6.2	72.5	50	10 T2DM participants	Glucose/3 h
864	^171^	United Arab Emirates (UAE)	Lulu (dates)	53.5	8.6	73.6	50	13 Healthy participants	Glucose/2 h
865	^171^	United Arab Emirates (UAE)	Lulu (dates)	43.8	7.7	73.6	50	10 T2DM participants	Glucose/3 h
866	^171^	United Arab Emirates (UAE)	Bo ma’an (dates)	46.3	7.1	72.7	50	13 Healthy participants	Glucose/2 h
867	^171^	United Arab Emirates (UAE)	Bo ma’an (dates)	51.8	6.9	72.7	50	10 T2DM participants	Glucose/3 h
868	^171^	United Arab Emirates (UAE)	Dabbas (dates)	49.1	3.6	76.2	50	13 Healthy participants	Glucose/2 h
869	^171^	United Arab Emirates (UAE)	Dabbas (dates)	50.2	3.9	76.2	50	10 T2DM participants	Glucose/3 h
870	^171^	United Arab Emirates (UAE)	Khalas (dates)	55.1	7.7	72.6	50	13 Healthy participants	Glucose/2 h
871	^171^	United Arab Emirates (UAE)	Khalas (dates)	53.0	6.0	72.6	50	10 T2DM participants	Glucose/3 h
872	^172^	United Arab Emirates (UAE)	Khalas (dates)	55.1	7.7	NA	50	13 Healthy participants	Glucose/2 h
873	^172^	United Arab Emirates (UAE)	Khalas (dates)	53.0	6.0	NA	50	10 T2DM participants	Glucose/3 h
874	^172^	United Arab Emirates (UAE)	Khalas with Arabic coffee	52.7	6.2	NA	50	13 Healthy participants	Glucose/2 h
875	^172^	United Arab Emirates (UAE)	Khalas with Arabic coffee	41.5	5.4	NA	50	10 T2DM participants	Glucose/3 h
876	^173^	United Arab Emirates (UAE)	Arabic bread	67	5	78.8	50	25 Healthy participants	Glucose/2 h
877	^173^	United Arab Emirates (UAE)	Regag bread	76	7	112.7	50	25 Healthy participants	Glucose/2 h
878	^173^	United Arab Emirates (UAE)	Chebab bread	54	8	109	50	15 Healthy participants	Glucose/2 h
879	^173^	United Arab Emirates (UAE)	Muhalla bread	77	2	73.9	50	15 Healthy participants	Glucose/2 h
880	^173^	United Arab Emirates (UAE)	Khameer bread	47	3	91.0	50	15 Healthy participants	Glucose/2 h
881	^173^	United Arab Emirates (UAE)	Fendal	74	7	158	50	20 Healthy participants	Glucose/2 h
882	^173^	United Arab Emirates (UAE)	Chami (cottage cheese)	60	9	470.0	25	16 Healthy participants	Glucose/2 h
883	^173^	United Arab Emirates (UAE)	Habba Hamra	47	3	313.3	50	15 Healthy participants	Glucose/2 h
884	^173^	United Arab Emirates (UAE)	Harees	42	2	323.0	50	15 Healthy participants	Glucose/2 h
885	^173^	United Arab Emirates (UAE)	Thareed (beef)	74	3	460.0	50	15 Healthy participants	Glucose/2 h
886	^173^	United Arab Emirates (UAE)	Biryani (chicken)	52	4	253.9	50	15 Healthy participants	Glucose/2 h
887	^173^	United Arab Emirates (UAE)	Machbous (fish)	60	3	277.0	50	20 Healthy participants	Glucose/2 h
888	^173^	United Arab Emirates (UAE)	Arseyah	72	4	507.6	50	15 Healthy participants	Glucose/2 h
889	^173^	United Arab Emirates (UAE)	Khabisa	67	4	89.1	50	15 Healthy participants	Glucose/2 h
890	^173^	United Arab Emirates (UAE)	Leqemat	44	4	113.1	50	15 Healthy participants	Glucose/2 h
891	^173^	United Arab Emirates (UAE)	Batheetha	59	4	130.8	50	20 Healthy participants	Glucose/2 h
892	^173^	United Arab Emirates (UAE)	Khanfaroosh	45	3	126.2	50	15 Healthy participants	Glucose/2 h
893	^173^	United Arab Emirates (UAE)	Balalet	63	5	179.3	50	15 Healthy participants	Glucose/2 h
894	^174^	United Arab Emirates (UAE)	Dried Bisr (mature unripe) dates	54.6	15.2	NA	25	15 Healthy participants	Glucose/2 h
895	^174^	United Arab Emirates (UAE)	Dried Tamr (mature ripe) dates	54.3	14.3	NA	25	15 Healthy participants	Glucose/2 h
896	^175^	Oman	Doughnut with water	75.49	3.44	250 (ml) water	50	12 Healthy participants	Glucose/2 h
897	^175^	Oman	Doughnut with Omani coffee	76.50	3.45	130 (ml) coffee	50	12 Healthy participants	Glucose/2 h
898	^175^	Oman	Croissant with water	67.46	2.47	250 (ml) water	50	12 Healthy participants	Glucose/2 h
899	^175^	Oman	Croissant with Omani coffee	65.32	3.69	130 (ml) coffee	50	12 Healthy participants	Glucose/2 h
900	^175^	Oman	Cheese sandwich with water	72.41	3.61	250 (ml) water	50	12 Healthy participants	Glucose/2 h
901	^175^	Oman	Cheese sandwich with Omani coffee	78.40	3.67	130 (ml) coffee	50	12 Healthy participants	Glucose/2 h
902	^175^	Oman	Chicken sandwich with water	65.68	3.00	250 (ml) water	50	12 Healthy participants	Glucose/2 h
903	^175^	Oman	Chicken sandwich with Omani coffee	70.61	3.66	130 (ml) coffee	50	12 Healthy participants	Glucose/2 h
904	^175^	Oman	Fried egg sandwich with water	73.38	4.46	250 (ml) water	50	12 Healthy participants	Glucose/2 h
905	^175^	Oman	Fried egg sandwich with Omani coffee	73.14	4.03	130 (ml) coffee	50	12 Healthy participants	Glucose/2 h
906	^175^	Oman	Sambosa vegetable with water	60.35	5.11	250 (ml) water	50	12 Healthy participants	Glucose/2 h
907	^175^	Oman	Sambosa vegetable with Omani coffee	57.25	3.97	130 (ml) coffee	50	12 Healthy participants	Glucose/2 h
908	^175^	Oman	Fried vermicelli with water	71.77	3.72	250 (ml) water	50	12 Healthy participants	Glucose/2 h
909	^175^	Oman	Fried vermicelli with Omani coffee	64.99	4.92	130 (ml) coffee	50	12 Healthy participants	Glucose/2 h
910	^175^	Oman	Boiled vermicelli with water	67.46	5.01	250 (ml) water	50	12 Healthy participants	Glucose/2 h
911	^175^	Oman	Boiled vermicelli with Omani coffee	64.02	5.26	130 (ml) coffee	50	12 Healthy participants	Glucose/2 h
912	^175^	Oman	Red bean with white bread and water	61.70	3.94	250 (ml) water	50	12 Healthy participants	Glucose/2 h
913	^175^	Oman	Red bean with white bread and Omani coffee	65.22	3.64	130 (ml) coffee	50	12 Healthy participants	Glucose/2 h
914	^176^	Saudi Arabia	Hassawi rice	59	5	150	25	13 Healthy participants	Glucose/2 h
915	^176^	Saudi Arabia	Uncle Ben’s rice	54	7	150	25	13 Healthy participants	Glucose/2 h
916	^177^	Saudi Arabia/United Kingdom	Khulas (dates)	55.0	6.0	NA	50	10 Healthy participants	Glucose/2 h
917	^177^	Saudi Arabia/United Kingdom	Khulas with Arabic coffee	63.0	5.0	NA	50	10 Healthy participants	Glucose/2 h
918	^178^	Saudi Arabia	Maktoomi	71.0	11.1	68.51	50	10 Healthy participants	Glucose/2 h
919	^178^	Saudi Arabia	Khudri	61.7	7.4	67.06	50	10 Healthy participants	Glucose/2 h
920	^178^	Saudi Arabia	Nabtat-ali	59.9	6.9	69.27	50	10 Healthy participants	Glucose/2 h
921	^178^	Saudi Arabia	Um-Kabar	58.7	7.3	69.11	50	10 Healthy participants	Glucose/2 h
922	^178^	Saudi Arabia	Ajwah	55.9	5.9	70.13	50	10 Healthy participants	Glucose/2 h
923	^178^	Saudi Arabia	Medjool	55.3)	6.8	70.54	50	10 Healthy participants	Glucose/2 h
924	^178^	Saudi Arabia	Sabaka	54.9	11.5	69.52	50	10 healthy participants	Glucose/2 h
925	^178^	Saudi Arabia	Ruthana	52.5	4	73.44	50	10 Healthy participants	Glucose/2 h
926	^178^	Saudi Arabia	Rashodia	50.9	6.5	67.27	50	10 Healthy participants	Glucose/2 h
927	^178^	Saudi Arabia	Wannanah	50.9	7.3	67.54	50	10 Healthy participants	Glucose/2 h
928	^178^	Saudi Arabia	Shishi	50.2	7.2	71.81	50	10 Healthy participants	Glucose/2 h
929	^178^	Saudi Arabia	Sukkary	43.4	4.7	77.63	50	10 Healthy participants	Glucose/2 h
930	^178^	Saudi Arabia	Shaqra	42.8	5.5	66.92	50	10 Healthy participants	Glucose/2 h
931	^179^	Lebanon	Bourgoul A banadoura	50.09	8.72	420	50	12 Healthy participants	Glucose/2 h
932	^179^	Lebanon	Fattit hommos	37.21	3.73	225	50	12 Healthy participants	Glucose/2 h
933	^179^	Lebanon	Loubieh bzet	12.76	4.90	240	50	12 Healthy participants	Glucose/2 h
934	^179^	Lebanon	Meghleh	49.50	7.69	390	50	12 Healthy participants	Glucose/2 h
935	^179^	Lebanon	Mehshe malfouf	67.93	7.89	525	50	12 Healthy participants	Glucose/2 h
936	^179^	Lebanon	Pizza	56.04	6.71	96	50	12 Healthy participants	Glucose/2 h
937	^179^	Lebanon	Riz A dgeg	57.34	6.67	600	50	12 Healthy participants	Glucose/2 h
938	^179^	Lebanon	Riz Bi halib	56.83	7.35	165	50	12 Healthy participants	Glucose/2 h
939	^179^	Lebanon	Sfouf	48.59	4.94	70	50	12 Healthy participants	Glucose/2 h
940	^179^	Lebanon	Siyadieh	14.62	3.24	430	50	12 Healthy participants	Glucose/2 h

*GI* glycaemic index, *NA* not available.

## Conclusion

We believe that the inclusion of the additional GI values of foods from non-Western countries will enhance the use and application of GI both in research and clinical practice. Many of the staples consumed in these regions are high in GI, notably rice, flatbread, noodles, buns, paus, pastries and so on. The use of these GI tables will also enable consumers to make informed choices on how best to select low GI foods. The GI data compiled in this article consists of both single and mixed meals. This is a major advance to many GI tables that have focused on single foods. Mixed meals in this region are complex in relation to ingredients used and taste. Given its complexity, our table that includes the GI of mixed meals is a major advantage. It is hoped that this compendium will further stimulate additional data collection and enhance the utility of GI tables for a worldwide audience.
